# Phosphorylation of TET2 by AMPK is indispensable in myogenic differentiation

**DOI:** 10.1186/s13072-019-0281-x

**Published:** 2019-06-04

**Authors:** Ting Zhang, Xiaowen Guan, Un Lam Choi, Qiang Dong, Melody M. T. Lam, Jianming Zeng, Jun Xiong, Xianju Wang, Terence C. W. Poon, Hongjie Zhang, Xuanjun Zhang, Hailin Wang, Ruiyu Xie, Bing Zhu, Gang Li

**Affiliations:** 1 Faculty of Health Sciences, University of Macau, Avenida da Universidade, Taipa, Macau; 2 Cancer Centre, Faculty of Health Sciences, University of Macau, Taipa, Macau; 3 National Laboratory of Biomacromolecules, Institute of Biophysics, Chinese Academy of Sciences, Beijing, 100101 China; 4 College of Life Sciences, University of Chinese Academy of Sciences, Beijing, 100049 China; 5 The State Key Laboratory of Environmental Chemistry and Ecotoxicology, Research Center for Eco-Environmental Sciences, Chinese Academy of Sciences, Beijing, 100085 China

**Keywords:** AMPK, TET2, Phosphorylation, Myogenesis, PAX7

## Abstract

**Background:**

TET-mediated oxidation of 5-mC participates in both passive and active DNA demethylation, which exerts a significant influence on diverse biological processes. Mass spectrometry has identified multiple phosphorylation sites of TET2. However, the functions of these phosphosites and their corresponding kinases are mostly unknown.

**Results:**

Here, we showed that AMP-activated protein kinase (AMPK) phosphorylates murine TET2 at the serine residue 97 (S97), and the phosphorylation enhances TET2 stability through promoting its binding to 14-3-3β. AMPK ablation resulted in decreased global 5-hmC levels at the myotube stages, severe differentiation defects of C2C12 cells and significantly, total loss of expression of *Pax7*. Genome-wide analyses revealed increased DNA methylation at genic and enhancer regions of AMPK-null myoblasts and myotubes. Using CRISPR/Cas9 technology, we showed that a novel enhancer, which is hypermethylated in AMPK-null cells, regulates *Pax7* expression. The phospho-mimicking mutant, TET2-S97E, could partly rescue the differentiation defect in AMPK-ablated C2C12 cells.

**Conclusions:**

Together, our data demonstrated that AMPK is a critical regulator of myogenesis, partly through phosphorylating TET2.

**Electronic supplementary material:**

The online version of this article (10.1186/s13072-019-0281-x) contains supplementary material, which is available to authorized users.

## Introduction

DNA demethylation is needed to reset epigenetic marks in development, particularly in the development of germ cells and early embryos [1–3]. It can be achieved through a passive DNA replication-dependent process or an active enzyme-catalyzed process. The active demethylation process is mainly mediated by ten-eleven translocation (TET) family proteins (TET1-3), which oxidize 5-methylcytosine (5mC) to 5-hydroxymethylcytosine (5hmC) and further down to 5-formylcytosine (5fC) and 5-carboxylcytosine (5caC) [4–6]. 5fC and 5caC can then be enzymatically excised by thymine-DNA glycosylase (TDG) and replaced with unmodified cytosine through base excision repair (BER) [7, 8].

Currently, our understanding of the mechanisms regulating TET proteins remains limited; we hypothesized that part of the answer lies in their post-translational modifications (PTMs). Indeed, many PTMs of TET proteins have been identified by high-throughput discovery-mode mass spectrometry and curated by PhosphoSitePlus (PSP, http://www.phosphosite.org/) [9]. However, for most of the PTMs of TET proteins, the enzymes that catalyze the PTMs remain to be identified and the corresponding biological functions remain largely unknown. In this study, we comprehensively examined the protein sequences of TET proteins and found TET2 harbors a well-defined substrate motif of AMP-activated protein kinase (AMPK). AMPK, as the central energy sensor of cells, is primarily activated by high AMP/ATP or ADP/ATP ratios. AMPK functions in diverse biological processes including energy homeostasis, embryonic growth and development, and its dysregulation are involved in the development of human diseases such as diabetes, obesity, inflammation and cancer [10–12]. Here, we show AMPK phosphorylates murine TET2 protein at the serine residue 97 (S97), and the phosphorylation stabilizes TET2 potentially through its increased binding to 14-3-3β, and provide evidence showing AMPK could exert its effect on epigenome through phosphorylating TET2, and be implicated in myogenesis.

## Results

### AMPK phosphorylates TET2 at Ser97 in vitro and in vivo

**Fig. 1:**
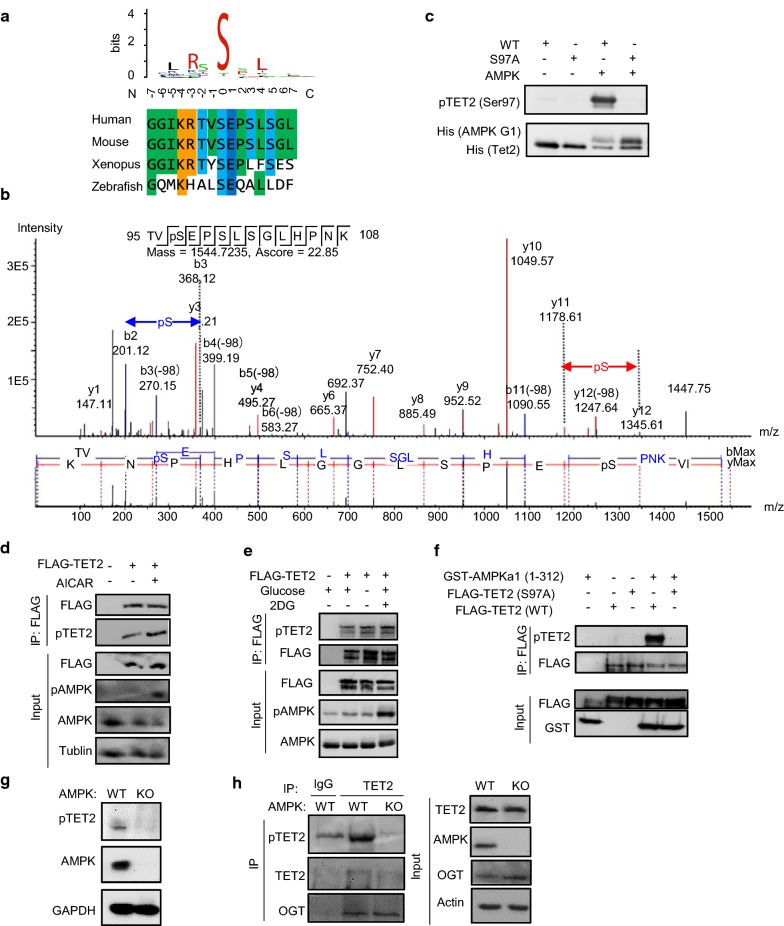
AMPK phosphorylates mouse TET2 at S97 in vitro and in vivo. **a** Murine TET2 harbors a well-established substrate motif of AMP-activated protein kinase (AMPK) around Ser 97. The logo motif of AMPK phosphorylation sites was generated by Web Logo [73] using data curated by Hardie et al. [14]. The residues surrounding S97 of murine TET2 which AMPK prefers are shown in red. The AMPK target motif around murine (S97) of TET2 is conserved across different species. **b** Phosphorylation of TET2 at Ser97 was detected in the product of an in vitro AMPK kinase reaction by mass spectrometry. **c** A phosphor-specific antibody against pSer97 of TET2 recognized wild-type TET2 phosphorylated by AMPK in vitro, but not a Ser97Ala (S97A) mutant. **d** Treatment with 5-aminoimidazole-4-carboxamide ribonucleotide (AICAR) induced TET2 phosphorylation. HEK293T cells transiently transfected with FLAG-tagged TET2 were treated with 2 mM AICAR for 24 h. FLAG-TET2 was immunoprecipitated with M2 beads (Sigma), and phosphorylation of TET2 was detected by Western blot analysis. **e** No glucose or 2-deoxy-d-glucose (2-DG) induced FLAG-TET2 phosphorylation. HEK293T cells were transiently transfected with FLAG-tagged TET2; cells were starved of glucose for 24 h or treated with 25 mM 2-DG for 2 h. FLAG-TET2 was immunoprecipitated with M2 beads (Sigma), and phosphorylation of TET2 was detected by Western blot analysis. **f** HEK293T cells were transiently transfected with FLAG-TET2 (WT or S97A) and GST-AMPKa1 (residues 1-312) for 24 h. FLAG-TET2 was immunoprecipitated with M2 beads (Sigma), and phosphorylation of TET2 was detected by Western blot analysis. **g** Knockout of AMPK diminished the phosphorylation of TET2 at Ser97. Expression of total TET2 and phosphor-TET2 (Ser97) in wild-type (WT) or AMPK knockout (KO) mouse embryonic fibroblasts (MEFs) cells was detected by Western blot analysis. **h** Phosphorylation of TET2 does not affect the interaction between TET2 and O-GlcNAc transferase (OGT). Immunoprecipitation of TET2 in wild-type (WT) or AMPK knockout (KO) mouse embryonic fibroblasts (MEFs) cells was followed by Western blot analysis using indicated antibodies. *a non-specific band

To identify the kinases targeting TET proteins, we first predicted the possible kinases for the known phosphorylation sites of TET proteins using *Scansite* [13] and found that murine TET2 harbors a well-defined substrate motif of AMP-activated protein kinase (AMPK) around Serine 97 [14]. This sequence motif is well conserved from fish to humans (Fig. 1a). To examine whether AMPK phosphorylates TET2 in vitro, we purified the recombinant N-terminus of murine TET2 (aa 1-181) (Additional file 1: Fig. S1) and performed in vitro kinase reaction. Mass spectrometry analysis of the reaction product revealed only one phosphorylation site, phosphoserine 97 (Fig. 1b). We next generated antibodies against phosphorylated TET2 (Ser97 [m], Ser99 [h]), enzyme-linked immunosorbent assay (ELISA) revealed that one of the antibodies (56012MR1) could distinguish the phosphorylated and non-phosphorylated peptides (Additional file 1: Table S1). We repeated the above in vitro kinase reaction and performed immunoblotting analysis using the antibody (56012MR1). As shown in Fig. 1c, the antibody did not cross-react with the unmodified TET2 protein. A signal was detected after in vitro AMPK kinase assay using the wild-type substrate. No signal was observed when the same kinase reaction was carried out using TET2 with serine 97 to alanine (S97A) mutation. This further indicated that AMPK phosphorylates TET2 at position S97, and the antibody (56012MR1) specifically recognizes TET2 phosphorylated at S97.

To test whether AMPK phosphorylates TET2 in vivo, we transfected a vector encoding FLAG-tagged TET2 into HEK293T cells and treated the transfected cells with 5-aminoimidazole-4-carboxamide ribonucleotide (AICAR) to induce AMPK activity. Immunoprecipitation followed by detection with the pTET2 (S97) antibody showed that phosphorylation of TET2 at S97 was increased after AMPK activation (Fig. 1d). Increased TET2 phosphorylation was also observed after activation of AMPK by no glucose culture, or by 2-deoxy-d-glucose (2-DG) treatment (Fig. 1e). To rule out that the increased TET2 phosphorylation was caused by non-specific effects of chemicals, we co-transfected full-length FLAG-tagged TET2 with a constitutively active form of AMPKα1 [15]. Immunoprecipitation followed by detection with pTET2 (S97) antibody clearly demonstrated the phosphorylation of TET2 by AMPK (Fig. 1f).

We then examined the phosphorylation of TET2 in immortalized wild-type (WT) or AMPKα1 and AMPKα2 double-knockout mouse embryonic fibroblasts (MEFs), and found that phosphorylation of TET2 at S97 significantly decreased in AMPK-null MEFs (Fig. 1g). To ascertain the result, we immunoprecipitated endogenous TET2 from WT and AMPK-null MEFs and observed a considerably diminished level of pTET2 (S97) in AMPK-null MEFs (Fig. 1h). These results indicate that AMPK is the physiological kinase for endogenous TET2 phosphorylation at Ser97 in vivo. However, the existence of residue phosphorylation of TET2 in the AMPK-null MEFs suggested that TET2 could be phosphorylated at S97 by other kinases too. To be noted, the interaction of TET2 and O-linked β-*N*-acetylglucosamine (GlcNAc) transferase (OGT) is not affected by the knockout of AMPK (Fig. 1h).

### AMPK activation enhances TET2 stability

**Fig. 2:**
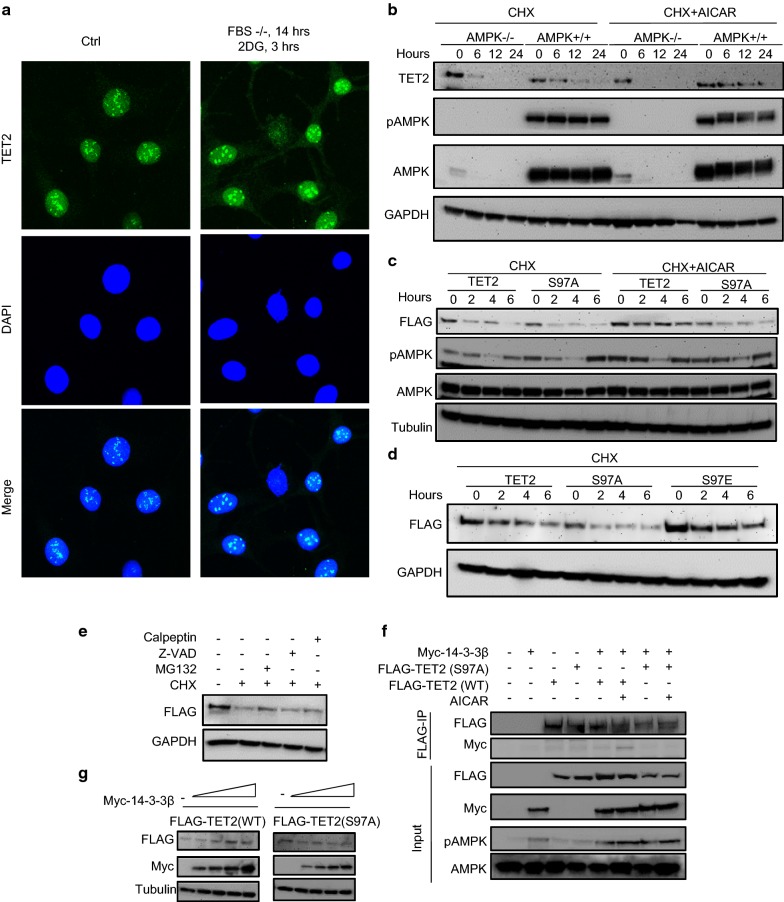
Phosphorylation of TET2 at Ser97 results in a stable TET2. **a** Mouse embryonic fibroblasts (MEFs) were serum starved for 14 h followed by treatment with 2-DG for 3 h. TET2 was detected by immunofluorescence staining (green). DAPI (4′,6-diamidino-2-phenylindole) was used to stain the cell nuclei (blue). **b** Wild-type or AMPK knockout mouse embryonic fibroblasts (MEFs) were treated with 100 µg/ml cycloheximide (CHX) alone, or in combination with AICAR for different periods. Levels of the indicated proteins are examined by immunoblotting analysis. GAPDH was used as a loading control. **c** Mutation of S97 to a non-phosphorylatable amino acid alanine (S97A) was sufficient to destabilize TET2. HEK293T cells were transfected with FLAG-tagged TET2 or FLAG-tagged TET2 S97A for 24 h; cells were then treated with 50 µg/ml CHX with or without 1 mM AICAR for different periods. Cell lysates were subjected to immunoblotting with the indicated antibodies. **d** Mutation of S97 to a glutamic acid that mimics phosphorylation of TET2 by AMPK results in a stable TET2. HEK293T cells were transfected with FLAG-tagged TET2, FLAG-tagged TET2-S97A or FLAG-tagged TET2-S97E for 24 h; cells were then treated with 50 µg/ml CHX for different periods. Cell lysates were subjected to immunoblotting with the indicated antibodies. **e** HEK293T cells were transfected with FLAG-TET2 for 24 h, then treated with 50 µg/ml cycloheximide (CHX) alone, or in combination with MG132 (10 µM), or Z-VAD-FMK (10 µM), or calpeptin (20 µM) for 12 h. Cell lysates were subjected to immunoblotting with the indicated antibodies. **f** 14-3-3β binds to phosphorylated TET2, but not to the S97A mutant. HEK293T cells were transiently transfected with the indicated constructs and treated with 1 mM AICAR for 3 h to induce AMPK activation. FLAG-TET2 was immunoprecipitated with M2 beads (Sigma), and Myc-14-3-3β was detected by Western blot analysis. **g** 14-3-3β binding stabilizes TET2. An increased amount of Myc-14-3-3β was co-transfected into HEK293T cells along with FLAG-TET2 or FLAG-TET2-S97A. Western blot analysis was performed to examine the expression of indicated proteins

To determine the functional effects of TET2 phosphorylation at S97, we first examined whether it will affect the subcellular localization of TET2. To this end, MEFs were cultured in serum-free medium for 14 h and then treated with 25 mM 2-DG for 3 h to activate the AMPK. Immunofluorescence staining of endogenous TET2 protein revealed that TET2 primarily localized in the nucleus, forming foci. Activation of AMPK promoted TET2 to form bigger and brighter foci in nuclei (Fig. 2a). Also, weak TET2 signal emerged in the cytoplasm after AMPK activation, indicating that activation of AMPK may drive the transfer of TET2 from the nucleus to the cytoplasm.

The increased TET2 immunofluorescence signals after AMPK activation promoted us to examine whether AMPK affects the stability of TET2 protein. We treated the MEFs with cycloheximide (CHX), which blocks protein synthesis, to determine TET2 stability by Western blot analysis. TET2 was apparently more stable in WT than in AMPK-null MEFs, and AICAR treatment, which induced AMPK activation, significantly increased the stability of TET2 in WT, but not in AMPK-null MEFs (Fig. 2b).

To determine whether the increased TET2 stability is achieved through phosphorylation at S97 by AMPK, we transiently transfected FLAG-tagged TET2, either in wild-type form (WT-TET2) or with a serine-to-alanine mutation at position 97 (TET2-S97A) mimicking the non-phosphorylated TET2, into HEK293T cells. The expression dynamics of FLAG-TET2 was followed after CHX treatment with or without AICAR treatment by Western blot analysis. As shown in Fig. 2c, TET2-S97A is not as stable as WT TET2, and AICAR treatment significantly increased the level of WT-TET2, but not TET2-S97A. In addition, increased accumulation of TET2 is observed when a serine-to-glutamic acid mutation was introduced at position 97 of TET2 (S97E) to mimic the Ser97-phosphorylation (Fig. 2d). These data suggested that AMPK-mediated phosphorylation of TET2 at S97 increased the stability of TET2 protein in cells.

To understand the underlying mechanisms of the enhanced TET2 stability induced by AMPK activation, we reexamined the potential degradation pathways of TET2. It has been reported that TET2 can be degraded by three different mechanisms: (a) the binding protein IDAX (CXXC finger protein 4) of TET2 can activate caspase 3 to degrade TET2 protein [16], (b) calcium-dependent protease calpain degrades TET1, TET2 and TET3 proteins [17]; (c) recently, Zhang et al. reported that TET2 protein is mainly degraded by ubiquitination-mediated pathways [18]. We repeated the relevant experiments to determine which TET2 degradation pathway is involved in our system. To this end, we used different proteolytic pathway inhibitors (MG132 (proteasome inhibitor); calpeptin (calpain inhibitor); Z-VAD-FMK (caspase inhibitor) to treat HEK293T cells overexpressing a FLAG-tagged form of TET2. Both of MG132 and calpeptin treatments increased the TET2 abundance after CHX treatment, indicating that TET2 is degraded by both ubiquitination-, and Calpain-mediated pathways (Fig. 2e).

By examining the amino acid sequence of TET2 protein, we found that the sequence encompassing Ser97 constitutes a motif that can be recognized by the 14-3-3 phosphorylated serine/threonine-binding proteins. Therefore, we hypothesized that 14-3-3 proteins might interact with TET2 and be involved in regulating its protein stability. To examine this hypothesis, FLAG-tagged TET2 protein and Myc-tagged 14-3-3β were co-expressed in HEK293T cells, and AMPK was activated by AICAR. Binding of wild-type TET2 to 14-3-3β was observed only when AMPK was activated; the mutant TET2 (TET2-S97A) did not bind to 14-3-3β under the same condition (Fig. 2f), indicating that the binding of TET2 to 14-3-3β is Ser97 phosphorylation-dependent. To investigate whether the binding of 14-3-3β to TET2 affects the stability of TET2, we co-transfected TET2 with different amounts of 14-3-3β and found that increasing the amount of 14-3-3β resulted in increased levels of TET2. However, the phenomenon is not seen in the TET2-S97A mutant (Fig. 2g), suggesting that the AMPK-induced TET2 stability is potentially achieved by an increase in the binding of TET2 and 14-3-3β.

### AMPK ablation resulted in severe differentiation defects of C2C12 cells

**Fig. 3:**
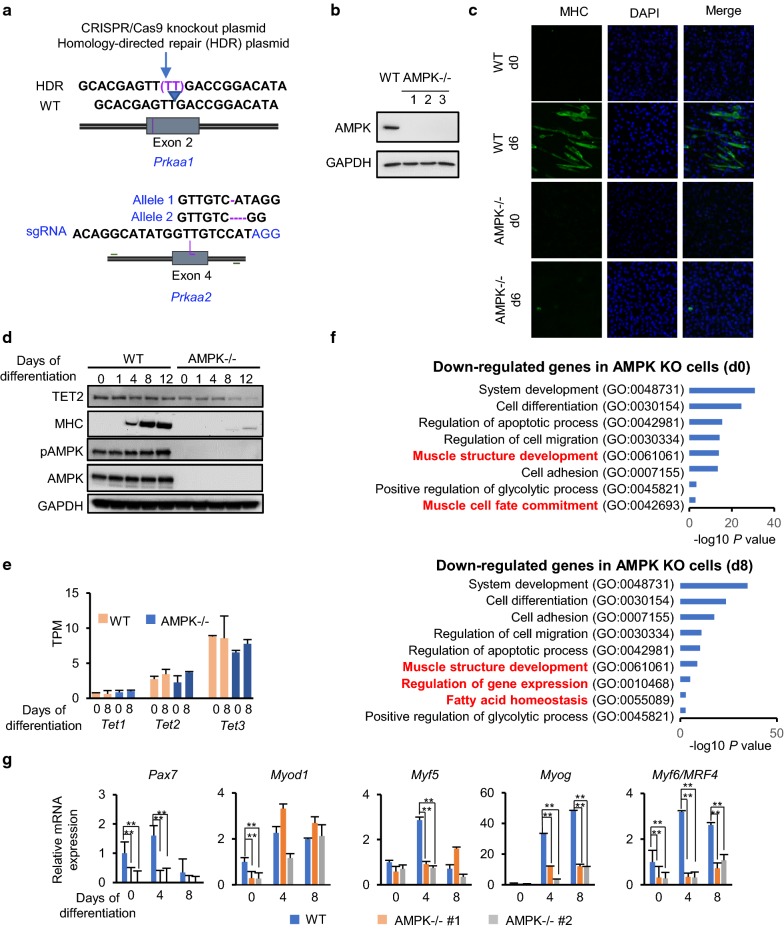
Knock-out of AMPK caused severe differentiation defect in C2C12 cells. **a** The strategy for knocking-out (KO) *AMPKa1* (*Prkaa1*) and *a2* (*Prkaa2*) in C2C12 cells. For knocking-out *AMPKa1* (*Prkaa1*), CRISPR/Cas9 knockout plasmid and homology-directed repair (HDR) plasmid from Santa Cruz were used. For knocking-out *AMPKa2* (*Prkaa2*), a sgRNA targeting exon 4 of *AMPKa2* (*Prkaa2*) [19] was used. Complete KO of *AMPKa1* and *a2* was confirmed by sequencing. The location of the two-base pair (TT) insertion in exon 2 of *Prkaa1* and deletions in exon 4 of *Prkaa2* alleles are indicated (purple dashed lines). **b** Knockout of AMPK in C2C12 cells was confirmed by Western blot analysis. GAPDH was used as a control. Shown are three independent clones of AMPK-KO. **c**, **d** Differentiation defects of AMPK-KO C2C12 cells. C2C12 cells were subjected to differentiation in 2% horse serum. MHC (Myosin heavy chain 1, MYH1) was detected by Western blot analysis (**c**) and immunofluorescence staining (**d**) during differentiation. Proteins examined are indicated. DAPI: 4′,6-diamidino-2-phenylindole. **e** Knockout of AMPK does not significantly change the expression of *Tet1*, *Tet2* and *Tet3* at mRNA levels in C2C12 cells. Expression data were retrieved from RNA sequencing data. TPM: transcripts per million. **f** Gene ontology analysis of downregulated genes in AMPK-KO C2C12 cells compared to wild-type cells at myoblast (differentiation d0) and myotube (differentiation d8) stages. **g** The expression of *Pax7* and myogenic regulatory factors (MRFs) was examined by RT-qPCR analysis. mRNA levels are presented relative to the levels in wild-type myoblasts (differentiation day 0) and were normalized to those of *Gapdh*. Data are presented as mean ± SD from three independent experiments performed in triplicates. *p < 0.05; **p < 0.01

To investigate the biological significance of phosphorylation of TET2 by AMPK, we knocked-out (KO) AMPK alpha 1 and alpha 2 by CRISPR-Cas9-mediated methods in C2C12 cells [19], resulting in multiple clones with AMPK deletions (Fig. 3a, b). By tracking changes in cell morphology and expression of myosin heavy chain (MHC) during C2C12 differentiation, a complete loss of differentiation potential toward myotubes was observed in the AMPK-/- cells (Fig. 3c, d and Additional file 1: Fig. S2). During the differentiation of C2C12 cells, the protein level of AMPK remained unchanged; however, the phosphorylation level of AMPK was slightly elevated on the 8th and 12th day of differentiation. A significant decrease in TET2 protein level was observed at the myoblast stage (day 0 of the differentiation) in the AMPK-deficient C2C12 cells. Although the protein levels of TET2 in WT C2C12 cells remained constant during differentiation, they were decreased in AMPK-deficient cells as the differentiation progressed, which was especially evident on the 8th and 12th day of differentiation (Fig. 3d).

Next, we analyzed the gene expression profiles of the C2C12 cells at myoblast (differentiation day 0) and myotube (differentiation day 8) stages by RNA-seq. The results showed that the mRNA level of *Tet1* was very low, and the level of *Tet3* was the highest, whereas *Tet2* was at an intermediate level in C2C12 cells (Fig. 3e). The absence of AMPK did not significantly affect the mRNA levels of *Tet1*, *2*, *3*, indicating that the observed decrease of TET2 protein in AMPK-deficient cells happened at the post-transcriptional level. Interestingly, the timing of the increase in AMPK phosphorylation during the differentiation of WT C2C12 cells matches the significant decrease of TET2 protein in AMPK-null cells, further suggesting the involvement of phosphorylation of TET2 by AMPK in regulating TET2’s protein stability.

A gene ontology analysis revealed that downregulated genes in AMPK-null C2C12 cells are involved in a wide range of biological processes, such as “system development,” “muscle structure development,” “muscle cell fate commitment” and others (Fig. 3f). Upregulated genes in AMPK-null C2C12 cells at the myotube stage are involved in “response to stress,” “metabolic process” and “regulation of cell size” (Additional file 1: Fig. S3). To further validate the results of RNA-Seq, we examined the expression of myogenic regulatory factors (MRFs) in two independent AMPK knockout C2C12 cell lines and WT C2C12 cells by RT-qPCR. As shown in Fig. 3g, *Myod1* and *Myf6/MRF4* were significantly downregulated in AMPK-deficient cells at the myoblast stage. At the myotube stage, the expression of *Myog* and *Myf6/MRF4* in AMPK-deficient cells decreased compared to WT cells. However, the expression of *Myod1* was comparable in between the WT and the AMPK-null cells at the myotube stage. Strikingly, a total loss of *Pax7* expression was observed in AMPK-null cells at both myoblast and myotube stages.

### Genome-wide DNA methylation changes in AMPK knockout C2C12 cells

**Fig. 4:**
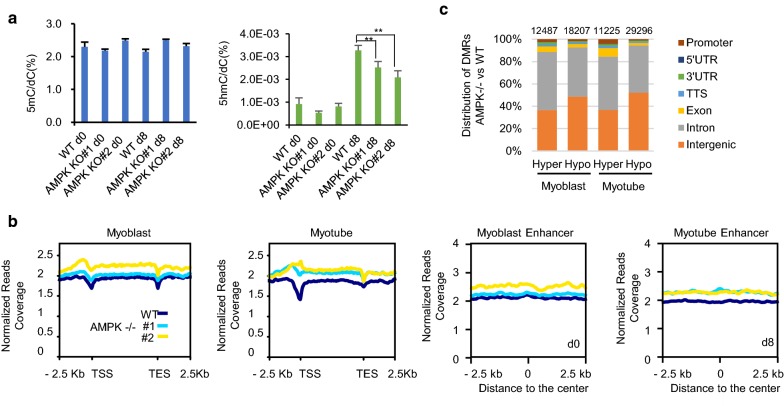
Genome-wide DNA methylation changes in AMPK knockout C2C12 cells. **a** Levels of 5mC (left) and 5hmC (right) in wild-type (WT) and AMPK knockout (KO) C2C12 cells at myoblast (d0) and myotube (d8) stages were detected by mass spectrometry. Two independent AMPK-KO lines were used. Data are presented as mean ± SD of triplicates. **p < 0.01. **b** Normalized methylated DNA immunoprecipitation sequencing (MeDIP-Seq) tag densities at genic and enhancer regions in wild-type (WT) and AMPK-/- C2C12 cells. Enhancers were defined according to their histone modification patterns [25]. 5mC enrichments from − 2.5 kb to + 2.5 kb relative to gene bodies or the centers of enhancers are shown. Two independent AMPK-KO lines were used. **c** The distribution of differentially methylated regions (DMRs) in AMPK-KO relative to wild-type C2C12 cells at myoblast (d0) and myotube (d8) stages across the genome. The number of hyper- or hypomethylated DMRs is shown on top of each bar

To investigate the effect of AMPK knockout on DNA methylation in C2C12 cells, we first measured the absolute content of 5-methylcytosine (5mC) and 5-hydroxymethylcytosine (5hmC) in C2C12 cells by liquid chromatography–tandem mass spectrometry (LC–MS/MS). As shown in Fig. 4a, global levels of 5mC did not change during the differentiation of C2C12 cells from myoblasts to myotubes, and AMPK knockout had no obvious effect on total 5mC content. However, an overall increase of 5hmC was observed during C2C12 differentiation, and the content of 5hmC was slightly decreased in AMPK-deficient myotubes, suggesting the potential involvement of AMPK in regulating 5hmC.

To examine changes in DNA methylation profiles caused by AMPK deficiency, we performed methylated DNA immunoprecipitation coupled with high-throughput sequencing (MeDIP-seq). Overall, 5mC signals showed a slight increase across gene bodies in the AMPK-deficient cells as compared to wild-type cells (Fig. 4b). In wild-type cells, the 5mC signal had a sharp dip at transcription start sites (TSSs), indicating that the TSSs are usually in a non-methylated state. In contrast, in AMPK-deficient cells, the dip of 5mC at the TSSs was significantly reduced, which was more pronounced when cells were differentiated into myotubes. Significantly, an upward peak of 5mC appeared upstream of the TSS in AMPK-deficient cells. Also, AMPK deficiency resulted in increased levels of methylation on the enhancers (Fig. 4b). Analysis of the MeDIP-seq data identified 12,487 hypermethylated differentially methylated regions (hyper-DMRs), and 18207 hypomethylated differentially methylated regions (hypo-DMRs), when comparing AMPK-deficient to WT C2C12 myoblasts (Fig. 4c). The hyper-DMRs of AMPK-deficient cells are enriched at the genic regions, including promoters, introns and exons. In contrast, hypo-DMRs tend to be enriched at the intergenic regions. This trend was more pronounced when cells were differentiated into myotubes, accompanied by a significantly increased number of hypo-DMRs. Remarkably, much more hyper-DMRs emerged in AMPK-deficient cells at the promoter and exon regions of target genes (Fig. 4c).

### An intragenic enhancer regulates *Pax7* expression

**Fig. 5:**
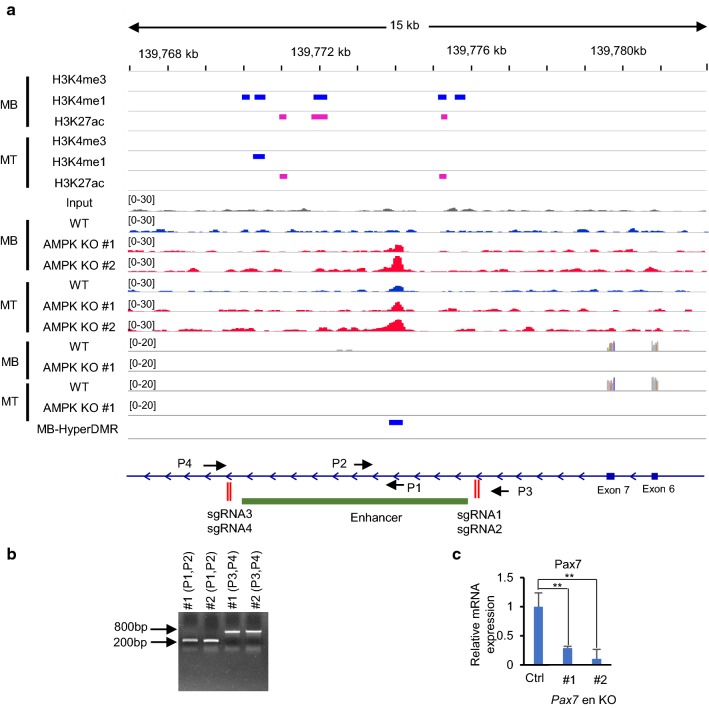
TET2 regulates *Pax7* expression through a putative enhancer located in intron 7 of *Pax7.* **a** Increased DNA methylation at a potential intragenic enhancer of *Pax7*. Normalized MeDIP-Seq and RNA-Seq tag densities in myoblasts (MB) and myotubes (MT) are shown. Peaks of H3K4me3 (MB: GSM628005; MT: GSM628006), H3K4me1 (MB: GSM1197187; MT: GSM1197187) and H3K27ac (MB: GSM921131; MT: GSM921133) were downloaded through cistrome DB. The locations of the Pax7 enhancer and sgRNA targeting sites for enhancer knockout are indicated. The myoblast-specific hyper-DMR in the intron 7 of *Pax7* is indicated in blue. Results from two independent AMPK-KO lines are shown. **b** PCR screening for CRISPR/Cas9-mediated deletion of *Pax7* intragenic enhancer in C2C12 cells. The locations of the PCR primers used for genotyping are displayed in panel (**a**). **c** The expression of *Pax7* in C2C12 clones with deletion of *Pax7* intragenic enhancer was examined by RT-qPCR. *Csnk2a* was used for normalization. Data are presented as mean ± SD from three independent experiments performed in triplicates. **p < 0.01

To elucidate why the AMPK deletion caused the striking disappearance of *Pax7* expression in C2C12 cells, we examined the DNA methylation statuses of the *Pax7* locus at the myoblast and myotube stages. According to the results of 5mC MeDIP, the DNA methylation statuses of the *Pax7* promoter and its upstream did not change significantly in AMPK-KO cells compared to WT cells. Instead, a significant hyper-DMR was found in the intron 7 of *Pax7* (Additional file 1: Fig. S4, Fig. 5a) in AMPK-KO C2C12 cells at the myoblast stage. To elucidate the potential function of this DMR, we downloaded all the ChIP-seq data deposited in the Gene Expression Omnibus (GEO) of multiple histone marks in C2C12 cells through the cistrome portal [20–22], including H3K4me3, H3K4me1 and H3K27ac [23–30] (Additional file 2: Table S2). Integrative analysis revealed a surprising finding: the *Pax7* intragenic DMR overlaps with a region with properties of active enhancers (Fig. 5a). To test whether this intragenic enhancer regulates the expression of Pax7, we knocked-out the enhancer by the CRISPR-Cas9-mediated method and obtained two independent clones containing a heterozygous deletion of the enhancer (Fig. 5b, Additional file 1: Fig. S5). RT-qPCR revealed that the deletion caused a significant decrease of *Pax7* expression in C2C12 cells (Fig. 5c), suggesting that this intragenic enhancer regulates *Pax7* expression.

### TET2 S97E partially rescues the differentiation defect of AMPK-null C2C12 cells

**Fig. 6:**
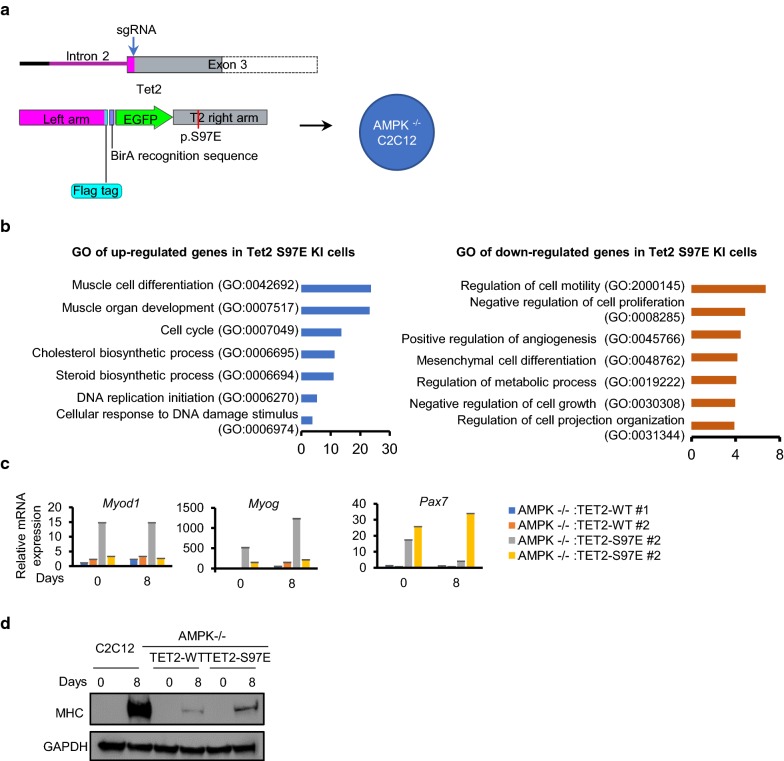
S97E mutation of TET2 partly rescues the differentiation defect of the AMPK-/- C2C12 cells. **a** The strategy for knocking in (KI) the p.S97E mutation of TET2 in AMPK-/- C2C12 cells. Upper panel: the target region. Lower panel: the HDR (homology-directed repair) donor. The c.289A > G;290G > A;291T > G mutation (red line) was introduced into the 3′ arm of exon 3 of the mouse TET2 gene to encode the phosphor mimic (serine-97-glutamic acid, S97E) of TET2. **b** Gene ontology (GO) analysis of differentially expressed genes (DEGs) between AMPK-/-:FLAG-BirA-TET2-S97E (TET2-S97E) and AMPK-/-:FLAG-BirA-TET2-WT (TET2-WT) C2C12 cells. **c** *Myod1*, *Myog* and *Pax7* mRNA contents were detected by RT-qPCR. Results of two independent C2C12 clones of AMPK-/-:FLAG-BirA-TET2-S97E (TET2-S97E) and AMPK-/-:FLAG-BirA-TET2-WT (TET2-WT) are shown. **d** Increased myosin heavy chain (MHC) expression after eight days induction of differentiation in AMPK-/-:FLAG-BirA-TET2-S97E (TET2-S97E) cells compared to AMPK-/-:FLAG-BirA-TET2-WT (TET2-WT) cells. Representative Western blot results are shown

AMPK regulates many aspects of cellular activities as an intracellular energy sensor. The observed phenotypes of the AMPK-deficient C2C12 cells, such as the inability to differentiate, abnormal DNA methylation status and loss of Pax7 expression, could be caused by other mechanisms than TET2-related. Hence, we next ask whether the phenotype of AMPK-/- C2C12 cells is TET2 phosphorylation-dependent. To this end, we knocked-in the S97E mutation along with FLAG-BirA tag and EGFP coding sequence to mimic TET2 in the S79-phosphorylated state at the endogenous TET2 locus in the AMPK-/- C2C12 cell through CRISPR-Cas9-mediated homologous recombination, whereas the same FLAG-BirA tag and EGFP coding sequence was knocked-in at the N-terminus of endogenous TET2 locus in the same background to serve as a control (Fig. 6a). We obtained multiple clones and verified that the knock-in was successful and homozygous (Additional file 1: Fig. S6), and named the cells carrying the S97E mutation as AMPK-/-:FLAG-BirA-TET2-S97E (TET2-S97E), the controls cells as AMPK-/-:FLAG-BirA-TET2-WT (TET2-WT). We performed RNA-seq on both cell lines at the undifferentiated states. Gene ontology (GO) enrichment analysis of the differentially expressed genes (DEGs) between S97E and WT cells revealed that genes involved in the biological process, such as “muscle cell differentiation,” “muscle organ development,” were upregulated in S97E cells (Fig. 6b). To further determine the effect of the S97E mutation on the expression of muscle differentiation-related genes, we examined the expression of *Myod1*, *Myog*, *Pax7* by RT-qPCR in two independent TET2-S97E and TET2-WT clones, respectively. The results showed that the expression of *Pax7*, *Myod1* and *Myog* was increased in TET2-S97E cells (Fig. 6c). Also, MHC expression was restored in TET2-S97E cells, albeit not to the levels of the wild-type cells, when cells were subjected to differentiation (Fig. 6d). The S97E mutation could not single-handedly rescue the differentiation defect of the AMPK-null C2C12 cells, as indicated by the still diminished level of MHC, and abnormal morphology of TET2-S97E cells at differentiation day 8 (Additional file 1: Figs. S7 and S8). Nonetheless, the increase in the expression of myogenic regulatory factors (MRF), especially in *Pax7*, is quite significant, which indicates that AMPK-mediated TET2 phosphorylation plays a vital role in myogenesis.

## Discussion

TET proteins are subject to substantial post-translational modifications (PTMs) [9, 31]; recent studies have started to elucidate the enzymes catalyzing these PTMs and their functions. For example, O-GlcNAc transferase (OGT) catalyzes the glycosylation of TET protein and affects gene transcription [32–35]. Monoubiquitylations of TET proteins by the E3 ubiquitin ligase complex CLR4(VprBP) result in enhanced binding of the TET protein to chromatin [36]. The acetylation of TET2 at the K110 residue by P300 enhances the enzymatic activity of TET2, while inhibiting the ubiquitination-mediated degradation of TET2 [18]. When this work was nearing completion, Wu et al. reported that AMPK phosphorylates human TET2 at S99, the same site as S97 in mouse TET2. They demonstrated that phosphorylation at S99 stabilizes TET2 by protecting it from calpain-mediated degradation, thereby elevates the level of 5hmC. They found that peripheral blood mononuclear cells (PBMCs) from patients with diabetes have lower levels of pAMPK, TET2pS99 and TET2 than healthy donors. Significantly, they demonstrated that TET2 is involved in glucose-modulated tumor growth, and AMPK activator metformin suppressed tumor growth partly by altering global 5hmC, through phosphorylating TET2 by AMPK [37].

Our results suggested that TET2 is probably degraded through multiple pathways, including the ubiquitin–proteasome pathway, and the calpain-mediated pathway. Our data indicated that 14-3-3ß binds to the S97-phosphorylated form of TET2, and S97A mutation abolishes the binding. Binding of 14-3-3ß to TET2 increased TET2 stability. However, the binding could have other effects on TET2, such as altering its localization, conformation and activity, which needs to be further examined. To be noted, the interaction between TET2 and OGT [32–34] remains intact in AMPK-null cells.

A striking finding of our study is that knockout of AMPK resulted in total loss of *Pax7* expression in C2C12 cells. PAX7, as a marker of adult muscle stem cells (MuSCs, also known as satellite cells) [38], is a transcription factor involved in the specification and maintenance of MuSCs. PAX7 binds to the promoters/enhancers of myogenic regulatory factors (MRFs), such as *Myod1* [39], *Myf5* [40] and *Myog* [41], to activate/prime their transcription in cultured myogenic cells. Mechanistically, PAX7 was proposed to work as a pioneer transcription factor to bind its targeted enhancers, triggering chromatin opening and DNA demethylation [41–43]. Therefore, it is possible that the differentiation defect may be mainly caused by the total loss of expression of *Pax7* in AMPK-deficient C2C12 cells.

Little is known about the transcriptional regulation of *Pax7* [44], and the enhancers for *Pax7* have not been fully characterized. Lang et al. generated three transgenic *LacZ* reporter mouse lines driven by 4 kb or 10 kb of genomic DNA upstream of the *Pax7* TSS, or the 4 kb of genomic DNA plus intron 1 genomic sequence. All these transgenic mice could not fully recapitulate the *Pax7* expression pattern during mouse development, conferring primarily neural expression instead [45], suggesting unknown enhancers for *Pax7* expression in MuSCs. Recently, Tichy et al. inserted the EGFP coding sequence in-frame immediately downstream of the first exon of *Pax7* based on a bacterial artificial chromosome, which contains continuous DNA sequence 81 kb upstream to 34 kb downstream of the *Pax7* locus. The resulting transgenic *Pax7EGFP* mouse recapitulates the expression of *Pax7* in MuSCs [46]. Our study demonstrated for the first time that a 6-kb region in the intron 7 of *Pax7* carried characteristics of an active enhancer, knockout of the region in C2C12 cells led to a significant down-regulation of *Pax7* expression, indicating that it is indeed an enhancer regulating *Pax7* in myoblasts.

Several previous studies have suggested the involvement of AMPK in the regulation of *Pax7* expression, but the results are somewhat conflicting. Theret et al. [47] reported that AMPKα1 deficiency in adult muscle stem cells (MuSCs) resulted in an increased number of PAX7-positive cells. In contrast, Fu et al. [48] reported that knockout of *AMPKα1* in MuSCs led to fewer PAX7-positive cells. Because of the dominant expression of *AMPKα1* in MuSCs, both studies only knocked-out *AMPKα1* in MuSCs. However, two paralogs exist for the AMPK α subunits, which compensates each other [49–52]. In this study, we showed double knockout of *AMPK α1* and *α2* in C2C12 led to an almost total loss of *Pax7* expression. Apparently, additional studies are needed to fully characterize the role of AMPK in the regulation of *Pax7* in MuSCs using AMPK α1 and α2 double-knockout animal models.

Dynamic changes in methylome occur during myogenesis and skeletal muscle adaption to various physiological conditions [53–55]. In this study, quantification of 5-hydroxymethylcytosine (5hmC) by LC–MS/MS revealed that 5hmC level increased during C2C12 myoblasts differentiation, validating a previous observation obtained by an immunostaining method [56]. We propose that the increase in 5hmC is partly due to the increased stability of phosphor-TET2 catalyzed by AMPK. Accumulated evidence suggests that metabolic signals play critical roles in shaping epigenome because many metabolites serve as substrates, cofactors or inhibitors of epigenetic modifying enzymes [57, 58]. Also, AMPK phosphorylates numerous downstream targets in responding to a variety of signals [10, 59]. Hence, other pathways might also contribute to the down-regulation of *Pax7* and severe differentiation defects in AMPK-null C2C12 cells. Considering AMPK phosphorylates FOXO3 and activates FOXO3’s transcriptional activity, it is plausible that AMPK regulates *Pax7* through the AMPK-FOXO3-NOTCH pathway [60]. However, the fact that the phospho-mimic form of TET2 partially rescued the phenotypes of AMPK-null C2C12 cells strongly argues that AMPK-mediated TET2 phosphorylation plays a critical role in myogenesis.

## Conclusions

Our findings demonstrate that AMPK acts on the epigenome (DNA methylation), partly through phosphorylating TET2, plays a crucial role in myogenesis. Notably, through regulating the methylation status of a novel enhancer of *Pax7*, AMPK is indispensable in maintaining the expression of *Pax7* in myoblasts. Whether AMPK has the same effect in MuSCs as in myoblasts requires further study. Nonetheless, our data presented here clearly demonstrated the importance of AMPK in myogenesis and revealed a potential mechanism for how AMPK alters epigenome. Reduced physical activity, aging, obesity and a variety of diseases including diabetes can lead to muscle atrophy, probably by affecting AMPK [61]. Hence, targeting AMPK has great potential to treat muscle atrophy.

## Methods

### Plasmids and Cloning

FH-Tet2-pEF was a gift from Anjana Rao (Addgene plasmid # 41710; http://n2t.net/addgene:41710; RRID:Addgene_41710). TET2 fragments were sub-cloned by PCR into the PET102 TOPO plasmid (Invitrogen) for bacterial expression. pX330-U6-Chimeric_BB-CBh-hSpCas9 was a gift from Feng Zhang (Addgene plasmid # 42230; http://n2t.net/addgene:42230; RRID:Addgene_42230). pEBG‐AMPKα1(1‐312) was a gift from Reuben Shaw (Addgene plasmid # 27632; http://n2t.net/addgene:27632; RRID:Addgene_27632). pcDNA3-myc3-14-3-3 Beta was a gift from Yue Xiong (Addgene plasmid # 19957; http://n2t.net/addgene:19957; RRID:Addgene_19957). Point mutations were introduced into the donor construct using the Quick Change II XL Site-Directed Mutagenesis Kit (Stratagene, CA).

### Recombinant protein purification

Escherichia coli BL21 (DE3) cells harboring the expression construct (pET102/D-TOPO vector) which encode the N-terminus of mouse TET2 (aa1-181) were grown in Luria–Bertani (LB) broth at 37 °C with vigorous shaking until the O.D.600 reaches 0.6. Isopropyl–D-thiogalactopyranoside (IPTG) was added to achieve a final concentration of 1 mM, and the culture was kept at 37 °C for 5.5 h with vigorous shaking. Cells were harvested, suspended in lysis buffer (NPI-10) containing lysozyme (1 mg/ml) and benzonase nuclease (3 units/ml culture volume) on ice for 30 min, and then centrifuged at 12,000 × *g* for 15–30 min at 4 °C. The supernatant was purified using Ni–NTA spin column (QIAGEN #31314). His-tagged purified recombinant wild-type TET2, or TET2 mutant (S97A) was used in the AMPK kinase assay.

### In vitro kinase assay and mass spectrometric analysis

Wild-type or S97A mutant form of recombinant His-tagged mouse TET2 protein (aa1-180) was incubated with AMPK A1⁄B1⁄G1 recombinant human protein (Thermo Fisher Scientific, PV4672) in the kinase reaction buffer: 50 mM Tris (pH 8.0), 10 mM MgCl2, 1 mM DTT, 1 mM EDTA, 0.3 mM NaCl and AMP (200 μM), with or without ATP (100 μM), at 30 °C for 1 h. The reaction was terminated with 2 × SDS buffer, separated by SDS-PAGE and subjected to Western blotting to detect TET2 phosphorylation using a phosphor-specific TET2 at Ser 97 (pTET2-S97) antibody, which was custom-made in this study. The reaction was also subjected to liquid chromatography–tandem mass spectrometry (LC–MS/MS) analysis to detect phosphorylation sites of TET2 at the Proteomics Core of the Faculty of Health and Science, the University of Macau.

### Western blot analysis and Immunoprecipitation

Cells were lysed in ice-cold lysis buffer (50 mM HEPES at pH 7.5, 150 mM NaCl, 1 mM EDTA, 1% NP-40, 10 mM pyrophosphate, 10 mM glycerophosphate, 50 mM NaF, 1.5 mM Na3VO4, 1 mM phenylmethylsulfonyl fluoride (PMSF), 1 mM dithiothreitol (DTT), protease inhibitor cocktail (Roche)) for 30 min and centrifuged at 12000 rpm for 15 min at 4 °C. For western blot analysis, an equal volume of 2 × Laemmli sample buffer was added to protein lysates and then heated up to 99 °C for 10 min for denaturing. Proteins were separated with SDS-PAGE electrophoresis, blotted onto nitrocellulose membranes (PALL). The membranes were blocked for 1 h at room temperature using 5% milk in PBST, and incubated with primary antibodies (Additional file 1: Table S3) overnight at 4 °C. The membranes were washed three times for 10 min each with PBST and incubated with the horseradish peroxidase (HRP) conjugated secondary antibodies in blocking buffer at room temperature for 1 h. The membranes were then washed three times for 5 min each with PBST, reacted with chemiluminescence reagents (Pierce™ ECL Western blotting substrate, Thermo Scientific), and the hybridization signals were captured using the iBright CL1000 imaging systems (ThermoFisher Scientific). For immunoprecipitation, the protein lysates were incubated with SureBeads™ protein G or A magnetic beads (Bio-Rad) for 1 h at 4 °C with gentle agitation to clear the lysates. The beads were collected with a magnetic rack then discarded. Cleared lysates were incubated with 1–3 µg antibodies overnight at 4 °C with gentle rotation. Magnetic beads were blocked with BSA for 1 h and washed twice in PBS, then added into the lysates. The lysate–beads mixture was then incubated for 4 h at 4 °C with gentle rotation. The pellet was collected and washed three times with BC300 buffer (20 mM Tris [pH 8], 0.3 M KCl, 10% glycerol, 1 mM EDTA, 1 mM dithiothreitol [DTT], 0.1% NP-40 plus proteinase inhibitors) and one time with BC100 buffer (20 mM Tris [pH 8], 0.1 M KCl, 10% glycerol, 1 mM EDTA and 1 mM DTT plus proteinase inhibitors). 2 × Laemmli sample buffer with β-mercaptoethanol was added to the pelleted beads, boiled for 8 min and analyzed using Western blotting.

### Cell culture, transfection and differentiation

Unless otherwise stated, cells were cultured at 37 °C in DMEM supplemented with 10% fetal bovine serum (Invitrogen) in an incubator with 5% CO2. For glucose starvation, cells were washed twice with 1 × PBS and then incubated in glucose- and pyruvate-free DMEM (Invitrogen) supplemented with 10% FBS. C2C12 cells were transfected using lipofectamine 2000 reagent (Invitrogen) or the 4D-Nucleofector™ System (Lonza) according to manufacturer’s instructions. HEK293T cells were transfected with polyethylenimine (PEI) in a 1:3 ratio (1 μg DNA : 3 μl PEI [1 mg/ml]). For in vitro differentiation, fully confluent C2C12 cells were washed twice with 1 × PBS and then subjected to differentiation in DMEM supplemented with 2% equine donor serum (Hyclone). Cells were harvested for RNA, DNA or protein extraction at desired time points.

### Stable cell lines

*Generation of AMPK knockout C2C12 cells* The CRISPR/Cas9 system was used to generate AMPKα1 and α2 knockout C2C12 cells. For knocking-out *AMPKα1* (*Prkaa1*), CRISPR/Cas9 knockout plasmid (sc-430618) and homology-directed repair (HDR) plasmid (sc-430618-HDR) from Santa Cruz were used. For knocking-out *AMPKα2* (*Prkaa2*), a previously published guide RNA sequence targeting exon 4 of *AMPKα2* (*Prkaa2*) was used [19]. Cells were transiently transfected with pX330 vectors containing guide RNAs along with a vector encoding puromycin resistance using the lipofectamine 2000 transfection reagent (Invitrogen #11668019). Two days after transfection, the cells were selected with 2 μg/ml puromycin for 48 h. Viable clones were grown to a larger size and picked up for Western blot analysis or sequencing.

*Knocking in the S97E mutation at the endogenous TET2 locus in C2C12 cells* The S97E mutation along with Flag-BirA tag was knocked-in at the endogenous TET2 locus through CRISPR-Cas9-mediated homologous recombination, whereas the same Flag-BirA tag was knocked-in at the N-terminus of endogenous TET2 locus in the same background to serve as a control. The homology-directed repair (HDR) donor construct containing the expression cassette of green fluorescent protein (GFP) and Flag-BirA tag, and a sgRNA construct targeting exon 3 of *Tet2* are gifts from Bing ZHU (Institute of Biophysics, Chinese Academy of Sciences) [62]. The S97E mutation was introduced into the donor construct using the Quick Change II XL Site-Directed Mutagenesis Kit (Stratagene, CA). Correct insertions were examined by PCR. Clones with biallelic insertions were kept, and the mutation was further validated by direct sequencing.

*Knocking*-*out the putative enhancer of Pax7 in C2C12 cells* To knock out the Pax7 enhancer, the CRISPR/Cas9 system was used with guide RNAs targeting the 5′ end (gRNA1, gRNA2) and 3′ end (gRNA3, gRNA4) of the Pax7 enhancer. C2C12 cells were transiently transfected with a mixture of pX330 vectors containing the gRNAs along with a vector encoding puromycin resistance using the lipofectamine 2000 transfection reagent (Invitrogen #11668019). Two days after transfection, the cells were selected with 2 μg/ml puromycin for 24 h. The cells were then subcultured at a low density allowing for individual clones to grow up. Clones with enhancer deletions were screened by PCR followed by gel electrophoresis. The enhancer deletion was further validated by direct sequencing.

### Immunofluorescence staining

Cells were seeded on coverslips coated with gelatin at an appropriate density. Cells were fixed by 4% paraformaldehyde for 10 min at room temperature and permeabilized with 0.2% Triton X-100 in PBS for 10 min at room temperature and blocked with the blocking buffer (1X PBS, 0.2% Triton, 3% BSA) for 30 min. Cells were then incubated with the primary antibody for 2-3 h at room temperature, washed three times with 0.2% Triton X-100 in PBS, then incubated with fluorescence-labeled secondary antibodies (Invitrogen, 1:500 dilution) for 30 min at room temperature and rewashed three times. A drop (10 μL) of VECTASHIELD antifade mounting medium containing 4′,6′-diamidino-2-phenylindole (DAPI) (Vector labs, H-1200) was added on a labeled microscope slide. The coverslip was then placed on the drop, observed and photographed under the Carl Zeiss LSM 710 confocal fluorescence microscope.

### RNA isolation and real-time PCR

Total RNA was isolated using RNeasy Mini kit (Qiagen). For the real-time RT-PCR, 1 μg RNA was reverse transcribed into cDNA in a 20-μL reaction using the PrimeScript™ RT Reagent Kit with gDNA Eraser (Takara). 1-μL diluted cDNA (20 ng) was used for real-time PCR in a 10-μL reaction consisted of 5-μL iTaq™ Universal SYBR^®^ Green Supermix (Bio-Rad), 1-μL (0.5 μM) gene-specific primers and water. Quantitative real-time PCR was performed in the CFX96 Real-Time Detection System (Bio-Rad), and reaction conditions were: 95 °C for 3 min, then followed by 40 cycles of denaturation at 95 °C for 5 s and annealing/extension at 60 °C for 30 s. Relative mRNA levels were calculated by normalizing to 18 s RNA or GAPDH mRNA. Primers used are described in Additional file 1: Table S4.

### DNA isolation

Cells were first resuspended in cell lysis buffer (20 mM Tris–HCl, 5 mM EDTA, pH 8.0), RNase and sodium dodecyl sulfate (SDS) were added afterward to a final concentration of 25 μg/ml and 0.125%, respectively, and the mixture was incubated at 37 °C for 5 h. Proteinase K was then added to a final concentration of 500 μg/ml followed by 8 h incubation at 65 °C. An equal volume of phenol–chloroform (1:1) was added, and the top aqueous phase was recovered. After that, 5 M NaCl was added to reach a final concentration of 0.4 M. The DNA was precipitated by adding 2 volumes of ethanol, washed twice with 70% ethanol and dissolved with ultrapure water.

### Liquid chromatography–mass spectrometry (LC–MS/MS) analysis of 5mC and 5hmC

Quantitative measurement of the absolute contents of 5mC and 5hmC by LC–MS/MS was performed as described previously [62]. Briefly, genomic DNA was digested into single nucleosides using DNase I, calf intestinal phosphatase and snake venom phosphodiesterase I. LC–MS/MS analysis was performed using G6410B triple quadrupole mass spectrometer with Agilent 1290 LC system (Agilent Technologies, CA). The frequency of 5mC and 5hmC was calibrated by spike-in standards of stable isotopic 5mC and 5hmC, respectively.

### Methylated DNA immunoprecipitation (MeDIP)

Genomic DNA was isolated by phenol–chloroform extraction as previously described. The MeDIP assay was carried out as recommended by the methylated DNA immunoprecipitation (MeDIP) kit (Abcam #ab117133). In brief, genomic DNA was sonicated to 200- to 600-bp. A total of 1 μg of the purified DNA fragments was used for the MeDIP reaction. MeDIP were performed with 1 µL of non-immune IgG or 1 µL of 5mC antibody (Abcam). The DNA was released by treatment with proteinase K and further purified for the library DNA preparation.

### MeDIP-Seq and RNA-Seq

Following 5mC MeDIP, MeDIP DNA libraries were generated according to the manufacturer’s instructions (NEB #E7645). Briefly, adaptor-ligated DNA was subjected to size selection using NEB Next Sample Purification Beads. Adaptor-ligated DNA was then subjected to PCR enrichment with adaptor primers for 10–12 cycles and then quantified using the Agilent 2100 Bioanalyzer (Agilent). The resulting DNA libraries were subjected to paired-end sequencing using the Illumina HiSeq 2000 platform (Illumina). For RNA-seq, total RNA was isolated according to the RNA extraction protocol of the Rneasy Mini kit (Qiagen). The constructed RNA-seq libraries were subjected to quality and purity check using Agilent 2100 Bioanalyzer (Agilent) and sequenced using the Illumina HiSeq 2000 platform in a 2 × 150 bp paired-end (PE) configuration.

### MeDIP-seq data processing and analysis

The quality of raw MeDIP-seq data was assessed with FastQC v0.11.7 (https://www.bioinformatics.babraham.ac.uk/projects/fastqc/), and adaptor sequences were trimmed using Trim Galore v0.4.5 (https://www.bioinformatics.babraham.ac.uk/projects/trim_galore/), with the stringency parameter set to 5 bp overlap. Reads were mapped to the mouse reference genome (USCS mm10 build) using Bowtie2 v2.3.4.1 [63] with default parameters. Duplicated reads were removed using MarkDuplicates of Picard tools v2.18.5 (http://broadinstitute.github.io/picard), and reads with map Q < 10 were filtered out using Samtools v1.3.1 [64]. To control for artifact signals, reads falling within regions listed on the ENCODE DAC blacklist (https://sites.google.com/site/anshulkundaje/projects/blacklists) were excluded in subsequent analyses.

The distribution of 5-mC was quantified by counting and normalizing mapped reads in each 50 bp bin with RPKM (reads per kilobase per million) using the “bamCoverage” function of Deeptools v3.0.2 [65]. Metagene profiles of 5-mC coverage around gene region and enhancer were plotted using the “computerMatrix” and “plotProfile” functions of Deeptools by mapping coverage data to the ± 2.5 kb region flanking the gene region (from TSS to TTS, scaled to 5 kb) or enhancer center at a resolution bin size of 50 bp. The coordinates of NCBI curated RefSeq genes (mm10) were downloaded from the UCSC Table Browser (https://genome.ucsc.edu/cgi-bin/hgTables) [66]. Enhancer regions for C2C12 myoblast and myotube were downloaded from Blum et al. [25] and converted to mm10 coordinates using the UCSC Tools—LiftOver (https://genome.ucsc.edu/cgi-bin/hgLiftOver).

Differentially methylated regions (DMR) in AMPK-KO with respect to WT were identified using the R-Bioconductor package—MEDIPS v1.30.0 [67] with an edgeR *p* value threshold of 0.05. The DMRs were then annotated with genomic features using the “annotatePeak” programme in HOMER v4.10 [68].

### RNA-seq data processing and analysis

The quality of sequencing reads was checked by FastQC, and Trim Galore was used to detect adapter contaminations at paired-end mode. Reads overlapping with adaptor sequences for at least 3 bp were trimmed, and low-quality reads were removed, keeping only reads longer than 36 bp. The cleaned reads were aligned to mouse reference genome GRCm38 (mm10) using STAR v2.5.3a [69] in a basic two pass mode for paired-end reads with the below parameters: –outReadsUnmapped None, –chimSegmentMin 12, –alignIntronMax 100000, –ChimSegmentReadGapMax parameter 3, –alignSJstitchMismatchNmax 5-1 5 5.

Gene expression was quantified using featureCounts v1.5.3 [70] with gene annotations (Release M12) downloaded from the GENCODE database [71]. Raw gene count was then normalized to TPM for expression comparisons between samples. For differential analysis, differentially expressed (DE) genes were identified using the R package—DESeq 2 v1.18.1 [72] with an adjusted p value threshold of 0.05. DE genes were functionally annotated with gene ontology (GO) using Metascape 3.0 (http://metascape.org).

## Additional files

**Additional file 1.** **Fig. S1.** SDS-PAGE analysis of the recombinant N-terminus of murine TET2 (aa 1-181) and its mutant. **Fig. S2.** AMPK knockout impaired the differentiation of C2C12 cells. **Fig. S3.** Gene ontology analysis of upregulated genes between AMPK-KO and wild-type C2C12 cells at myoblast- (differentiation d0, A) or myotube- stage (differentiation d8, B). **Fig. S4.** Increased DNA methylation at a potential intragenic enhancer of Pax7. **Fig. S5.** CRISPR/Cas9-mediated deletion of the Pax7 intragenic enhancer in C2C12 cells. **Fig. S6.** Knocking in (KI) the pS97E mutation of Tet2 in AMPK-/- C2C12 cells. **Fig. S7.** S97E mutation of TET2 partly rescues the differentiation defect of the AMPK-/- C2C12 cells. **Fig. S8.** Increased myosin heavy chain (MHC) expression in AMPK-/- C1C12 cells rescued with TET2 harboring S97E. **Table S1.** ELISA analysis of pTET2 [Ser99 (h); Ser97(m)] antibodies*. **Table S3.** Antibodies. **Table S4:** Primers and Oligos.

**Additional file 2.** **Table S2.** Peaks of Histone marks at the mouse Pax7 locus* (Excel file).

## Data Availability

All sequencing data are deposited in the Gene Expression Omnibus (GEO) database under the Accession number GSE126371.
