# Supplementary material for: Phosphorylation of TET2 by AMPK is indispensable in myogenic differentiation
Source: Epigenetics Chromatin. 2019 Jun 4;12:32. doi: 10.1186/s13072-019-0281-x (PMC6547497; doi:10.1186/s13072-019-0281-x)

Additional file information

Phosphorylation of TET2 by AMPK is indispensable in myogenic differentiation

Ting Zhang[1,2], Xiaowen Guan[1,2], Un Lam Choi[1,2], Qiang Dong[3], Melody M. T. Lam[1,2], Jianming Zeng[1,2], Jun Xiong[3], Xianju Wang[1,2], Terence C.W. Poon[1,2], Hongjie Zhang[1,2], Xuanjun Zhang[1,2], Hailin Wang[5], Ruiyu Xie[1,2], Bing Zhu[3,4], Gang Li[1,2]*

[1] Faculty of Health Sciences, University of Macau, Avenida da Universidade, Taipa, Macau.

[2] Cancer Centre, Faculty of Health Sciences, University of Macau, Taipa, Macau.

[3] National Laboratory of Biomacromolecules, Institute of Biophysics, Chinese Academy of Sciences, Beijing 100101, China.

[4] College of Life Sciences, University of Chinese Academy of Sciences, Beijing 100049, China.

[5]The State Key Laboratory of Environmental Chemistry and Ecotoxicology, Research Center for Eco-Environmental Sciences, Chinese Academy of Sciences, Beijing 100085, China.

*Correspondence: gangli@um.edu.mo (G.L.)

Contents:

Additional Figures S1-S8

Additional Tables (Table S1-S4)

Full Length Gels and Blots

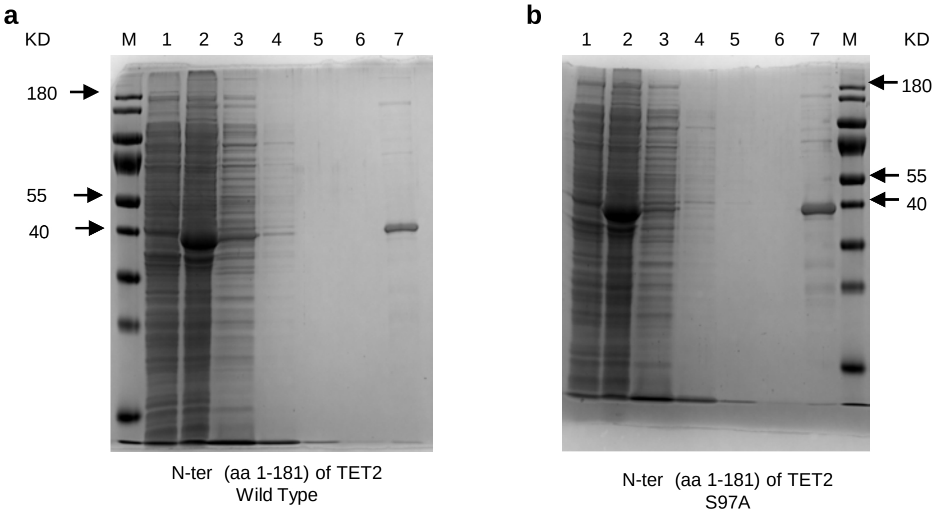

Figure S1

SDS-PAGE analysis of the recombinant N-terminus of murine TET2 (aa 1-181) and its mutant. a & b, Lane1, 500 µl bacteria (BL21) before induction of protein expression were pelleted by centrifugation and lysed in 60 µl 1 × SDS sample buffer; 10 µl was loaded; Lane 2, protein expression was induced with 0.1 mM IPTG at 37 °C for 5.5 h, bacteria were lysed with the NPI-10 lysis buffer (Qiagen); Lane 3, first flow through; Lane 4, first wash; Lane 5, second wash; Lane 6, third wash; Lane 7, purified wild-type or S97A mutant recombinant TET2 (aa 1-181). M, protein molecular weight markers. The gel was stained with Coomassie Blue R-250.

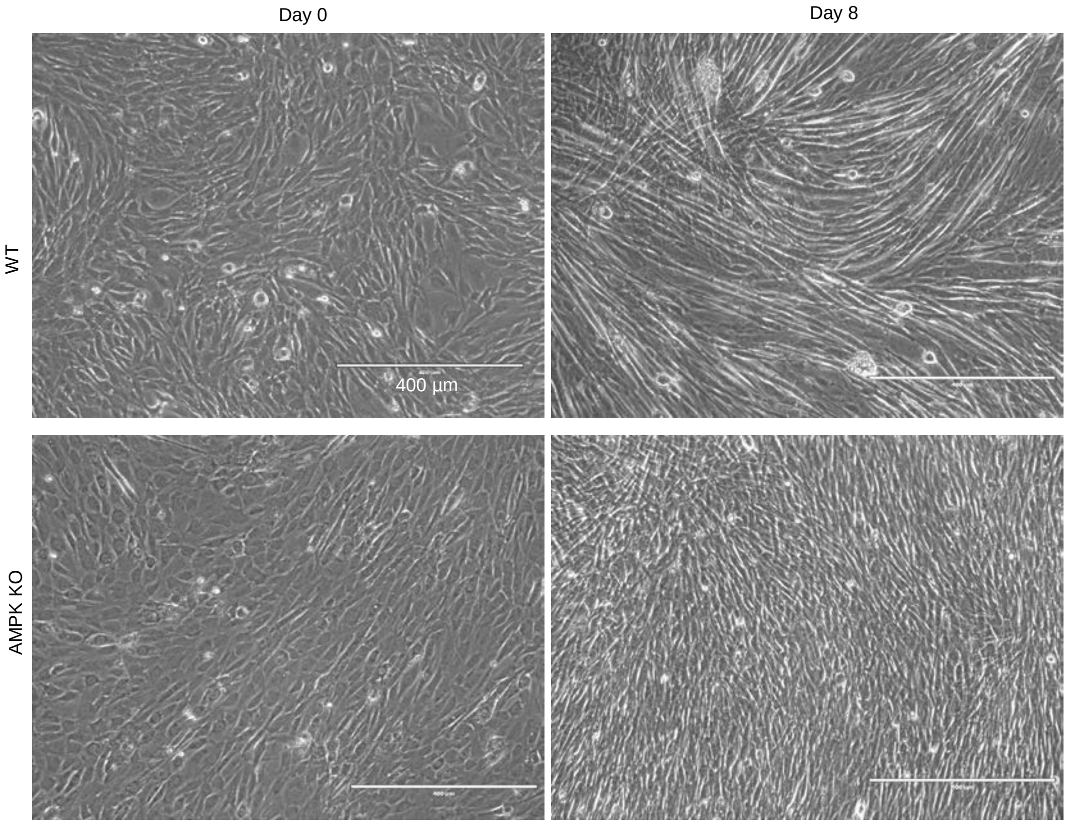

Figure S2

AMPK knockout impaired the differentiation of C2C12 cells. Shown are phase contrast images of wild-type (WT) and AMPK knockout (KO) C2C12 cells at day 0, or day 8 of differentiation in 2% horse serum.

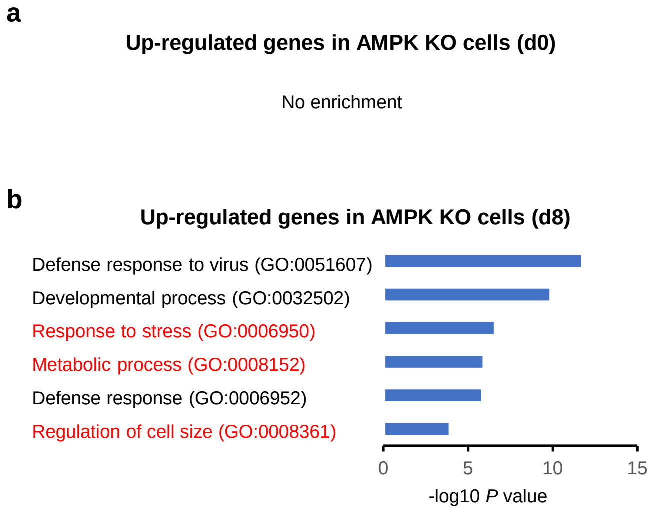

Figure S3

Gene ontology analysis of up-regulated genes between AMPK-KO and wild-type C2C12 cells at myoblast- (differentiation d0, **a**) or myotube- stage (differentiation d8, **b**).

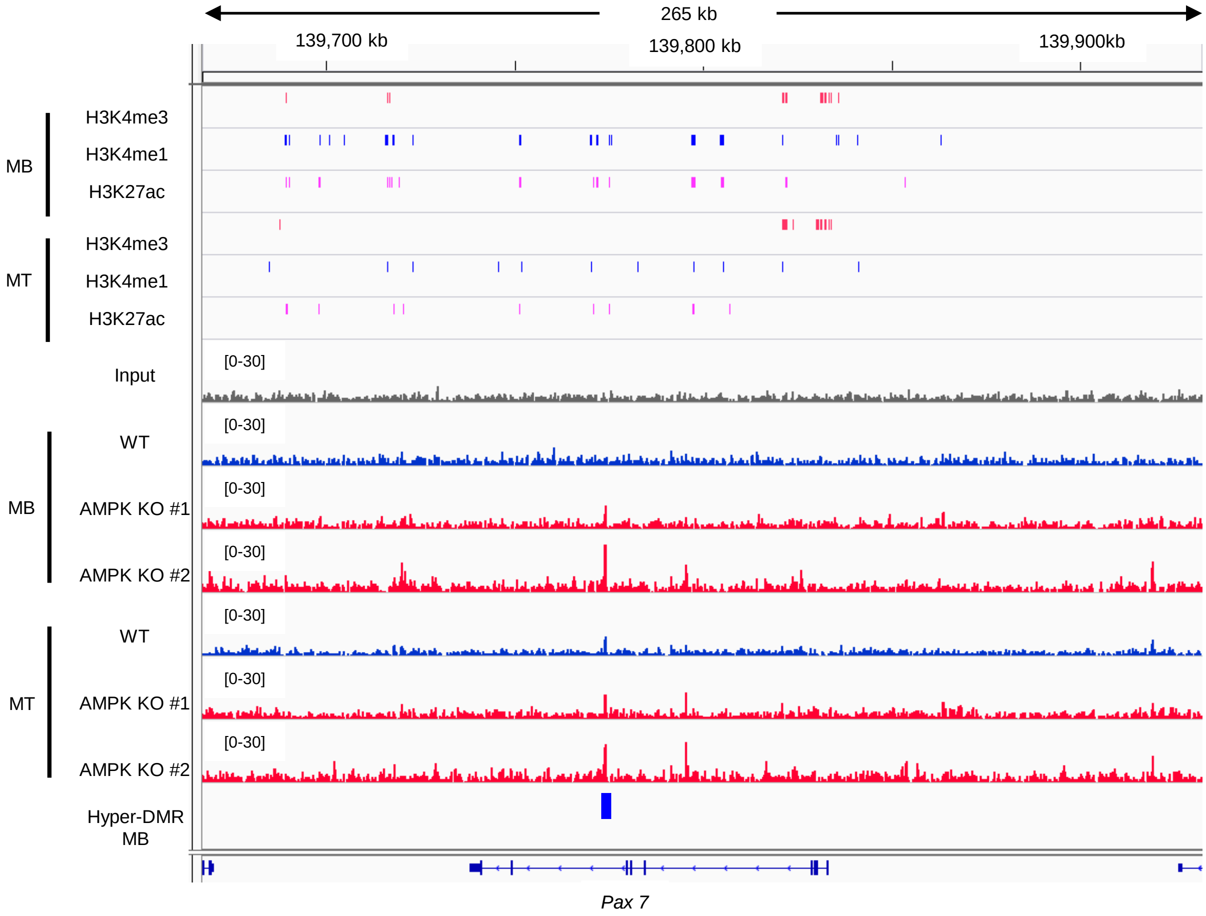

Figure S4

Increased DNA methylation at a potential intragenic enhancer of *Pax7*. Normalized MeDIP-Seq tag densities in myoblasts (MB) and myotubes (MT) are shown. Peaks of H3K4me3 (MB: GSM628005; MT: GSM628006), H3K4me1 (MB: GSM1197187; MT: GSM1197187) and H3K27ac (MB: GSM921131; MT: GSM921133) were downloaded through CistromeDB. The myoblast-specific hyper-DMR at the intron 7 of *Pax7* is indicated in blue.

a

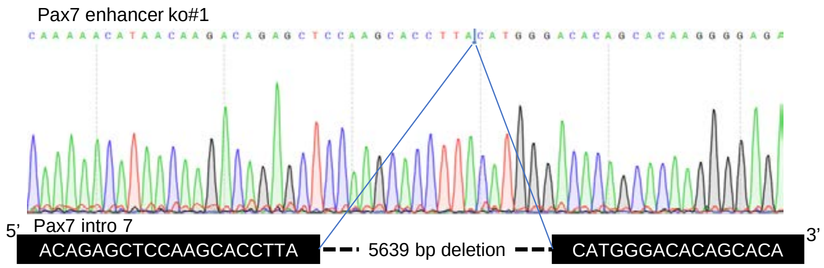

b

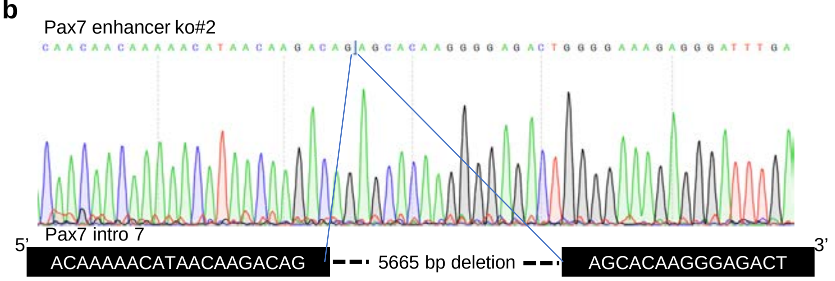

Figure S5

CRISPR/Cas9 mediated deletion of the *Pax7* intragenic enhancer in C2C12 cells. a & b, The sequencing chromatograms of the region harboring the *Pax7* intragenic enhancer. The results from two independent clones of *Pax7* enhancer knockout are shown. The sequences of PCR products were determined by Sanger sequencing. Dashed lines indicate the deleted genomic regions, 5639 bp and 5665 bp fragments were deleted in clone #1 and clone #2, respectively.

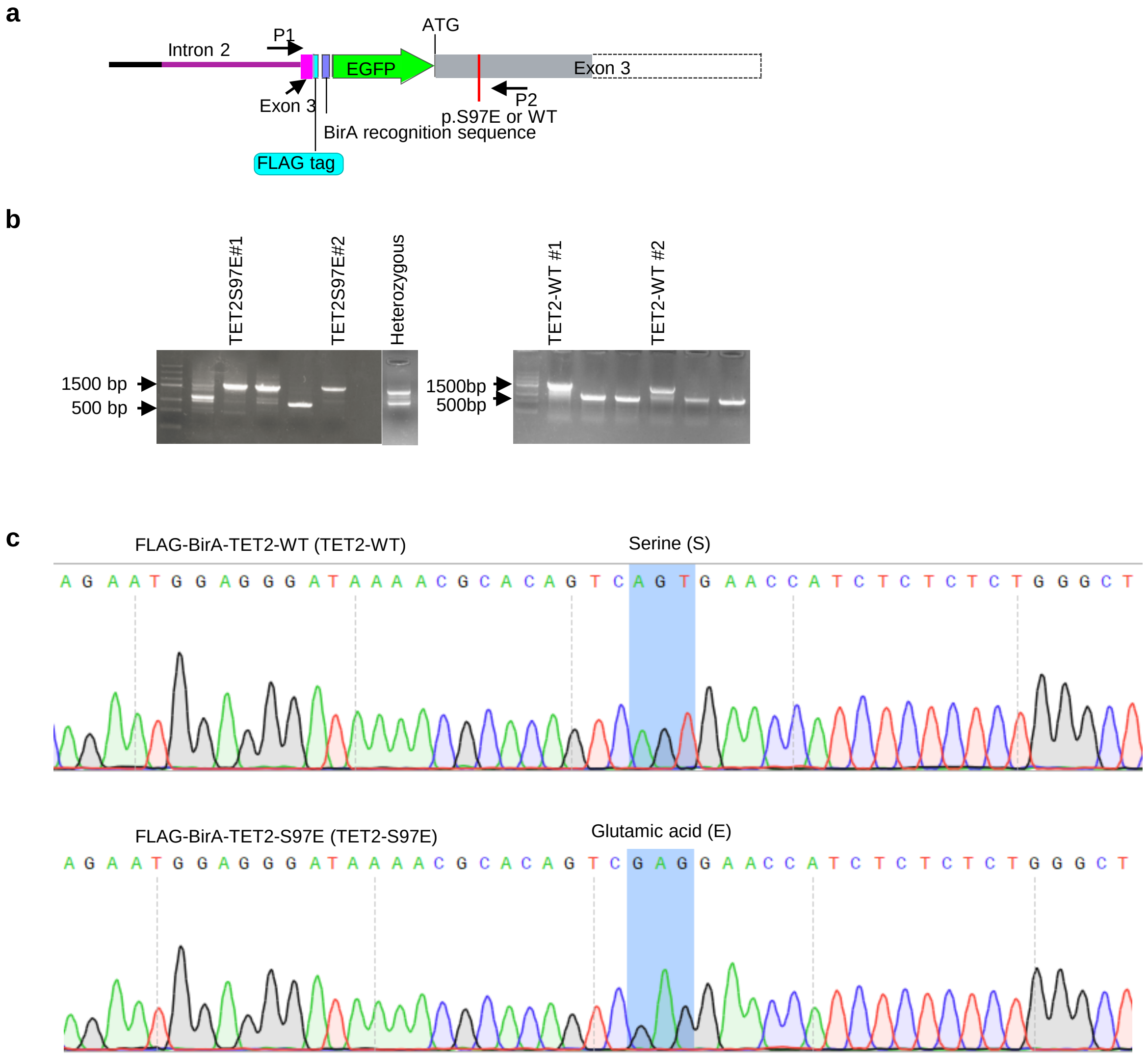

Figure S6

Knocking in (KI) the pS97E mutation of *Tet2* in AMPK-/- C2C12 cells. a, A diagram for the knockin allele. The FLAG-BirA tag and enhanced green fluorescent protein (EGFP) coding sequence are inserted into exon 3, in front of the start codon. The 5' homology arm is indicated in purple and 3' homology arm is indicated in grey. The c.289A>G;290G>A;291T>G (pS97E) mutation is indicated by a red line. The primers (P1, P2) used for screening and the start codon are shown. **b,** PCR screening of the homozygous knockin of the pS97E mutation or wild-type *Tet2* at the endogenous locus in AMPK-/- C2C12 cells. The expected size of the PCR products from successful knockin clones is 1650 bp. **c,** The sequencing chromatograms of the region harboring the S97 coding site of *Tet2*. The sequences of PCR products were determined by Sanger sequencing.

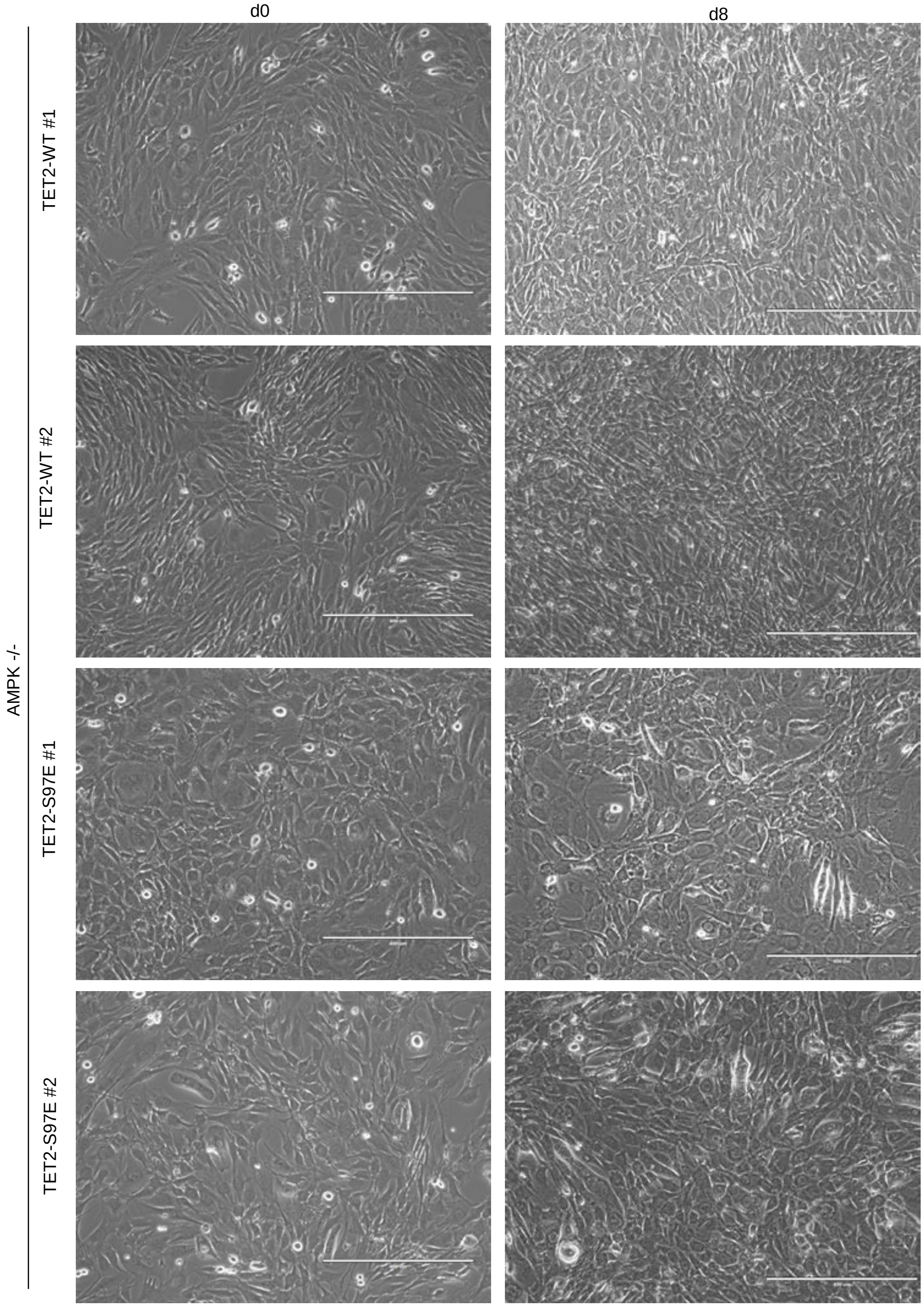

Figure S7

S97E mutation of TET2 partly rescues the differentiation defect of the AMPK-/- C2C12 cells. Shown are phase contrast images of AMPK-/-: FLAG-BirA-TET2-Wild Type (TET2-WT) cells and AMPK-/-: FLAG-BirA-TET2-S97E (TET2-S97E) cells before differentiation (d0) or, or after 8 days of differentiation in 2% horse serum (d8).

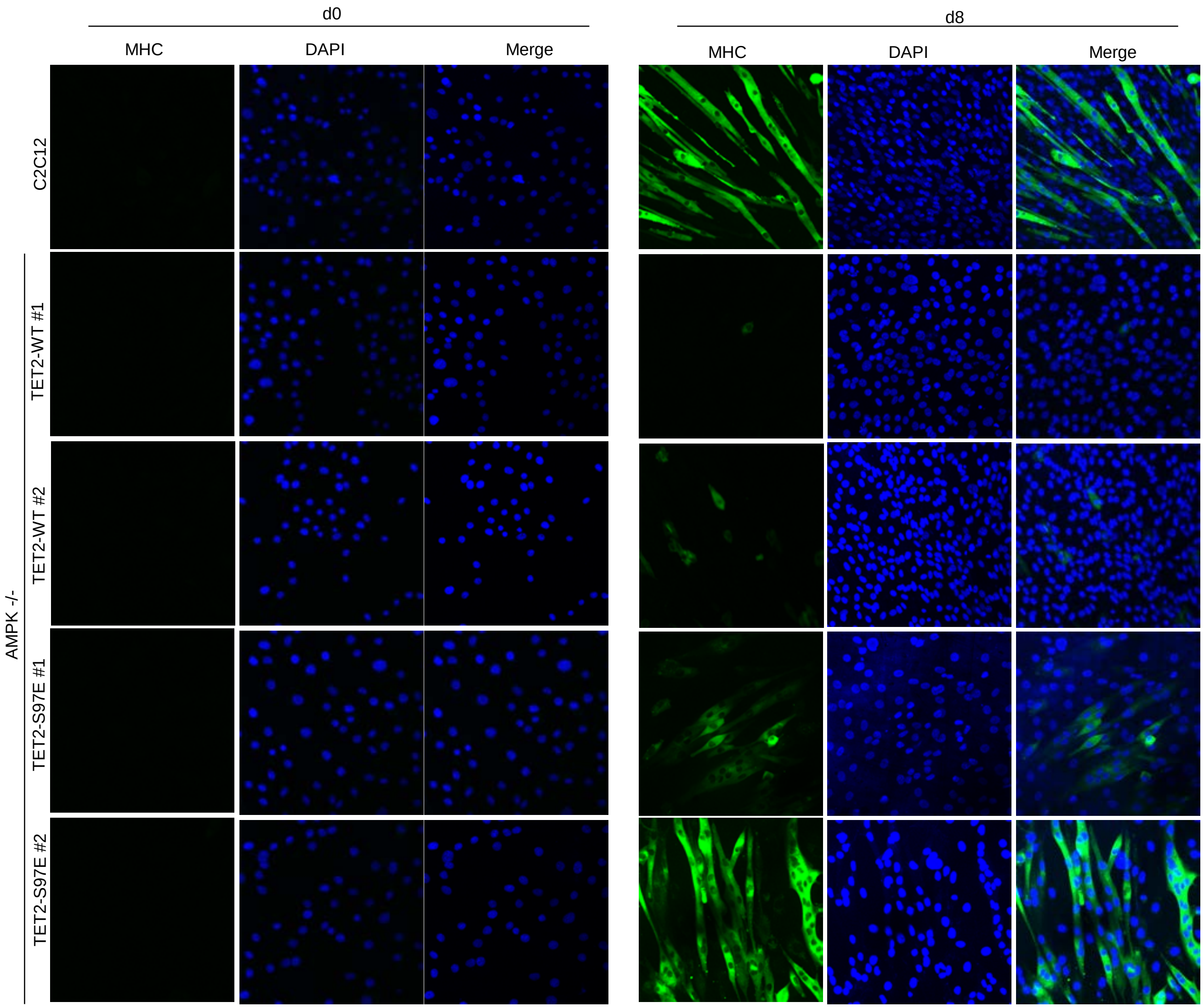

Figure S8

Increased myosin heavy chain (MHC) expression in AMPK-/- C1C12 cells rescued with TET2 harbouring S97E. AMPK-/-: FLAG-BirA-TET2-S97E (TET2-S97E) cells and AMPK-/-: FLAG-BirA-TET2-Wild Type (TET2-WT) cells were subjected to differentiation in 2% horse serum. MHC was detected by immunofluorescence and the nuclei were stained with 4',6-diamidino-2-phenylindole (DAPI). Two independent clones for each line and the parental C2C12 cells are examined.

Table S1. ELISA analysis of pTET2 [Ser99 (h); Ser97(m)] antibodies*

Antibody	1250	2500	5000	10000	20000	40000	80000	IgG	Antigen
**560-1-2-M-R1**	**3.084**	**3.077**	**2.903**	**2.511**	**2.274**	**1.876**	**1.182**	**0.064**	
560-1-2-M-R3	3.089	2.783	2.572	2.233	1.784	1.162	0.674	0.069	M
560-1-2-C	3.064	3.078	3.017	2.984	2.887	2.426	2.078	0.067	
560-1-2-M-R1	0.164	0.133	0.102	0.083	0.075	0.07	0.072	0.073	
560-1-2-M-R3	0.085	0.078	0.071	0.067	0.068	0.07	0.068	0.069	C
560-1-2-C	3.073	2.892	2.71	2.281	1.84	1.174	0.678	0.07	

*Note: Phospho-specific antibodies against TET2 phosphorylated at Ser99 (h) or Ser97(m) were produced by immunizing rabbits with a phospho-peptide (IKRTV-pS-EPSLSGL), which exists in both of human and murine TET2. As indicated by the enzyme-linked immunosorbent assay (ELISA) results, 56012MR1 possesses the ability to distinguish phosphorylated (M) and non-phosphorylated peptide (C).

Table S3. Antibodies

Antibody Name	Supplier and Cat. Number	Dilution	Usage
AMPKα (D5A2) Rabbit mAb	Cell signaling technology no.5831	1:1000	Western blot
Phosphor-AMPK α (Thr 172)	Cell signaling technology #2535	1:1000	Western blot
Rabbit anti- ACC	Cell signaling technology #3676	1:1000	Western blot
Phospho-Acetyl-CoA Carboxylase (Ser79)	Cell signaling technology #3661	1:1000	Western blot
14-3-3 (pan)	Cell signaling technology #8312	1:1000	Western blot
Phospho-(Ser) 14-3-3 Binding Motif	Cell signaling technology #9601	1:1000	Western blot
GAPDH (D16H11) XP® Rabbit mAb	Cell signaling technology #5174	1:5000	Western blot
Myc-Tag (9B11) Mouse mAb	Cell signaling technology #2276	1:1000	Western blot
Rabbit anti-TET2	Abcam #94580	1:1000	Western blot
Anti-6X His tag	Abcam #ab18184	1:1000	Western blot
Rabbit anti-OGT	Abcam #ab177941	1:1000	Western blot
Anti-Vinculin	Abcam #ab18058	1:1000	Western blot
Anti-alpha Tubulin	Abcam #ab7291	1:1000	Western blot
β-Actin Antibody (C4)	Santa Cruz #sc-47778	1:1000	Western blot
Myosin Heavy Chain Antibody (MF20)	R&D #MAB4470	1:1000	Western blot
		1:100	Immunofluorescence

Mouse anti-TET2	Active Motif #61389	1:1000	Western blot
5-Hydroxymethylcytosine (5-hmC)	Active Motif #39791	1:10000	Dot blot, hMeDIP
5-Methylcytosine (5-mC) antibody	Active Motif #39649	1:10000	Dot blot, MeDIP
Anti-FLAG M2-peroxidase (HRP)	Sigma #A8592	1:1000	Western blot
Mouse anti-FLAG-M2	Sigma #F1804	1:1000	Western blot
Rabbit anti-pTET2 (S97)	Custom made	1:1000	Western blot
Peroxidase-AffiniPure Goat Anti-Mouse IgG (H+L)	Jackson Immuno Research #115-035-146	1:5000	Western blot
Peroxidase-AffiniPure Goat Anti-Rabbit IgG (H+L)	Jackson Immuno Research #111-035-144	1:5000	Western blot
Peroxidase-AffiniPure Goat Anti-Mouse IgG, Light Chain	Jackson Immuno Research #115-035-174	1:5000	Western blot
Peroxidase-IgG Fraction Monoclonal Mouse Anti-Rabbit IgG, Light Chain	Jackson Immuno Research #211-032-171	1:5000	Western blot
Peroxidase-AffiniPure Goat Anti-Rabbit IgG, Fc Fragment Specific	Jackson Immuno Research #111-035-046	1:5000	Western blot
AffiniPure Goat Anti-Mouse IgG, Fcγ Fragment Specific	Jackson Immuno Research #115-005-071	1:5000	Western blot

Goat anti-Mouse IgG (H+L) Secondary Antibody, Alexa Fluor® 594 conjugate	Invitrogen #A-11005	1:500	Immunofluorescence
Goat anti-Rabbit IgG (H+L) Secondary Antibody, Alexa Fluor® 594 conjugate	Invitrogen #A-11037	1:500	Immunofluorescence
Goat anti-Rabbit IgG (H+L) Secondary Antibody, Alexa Fluor® 488 conjugate	Invitrogen #A11034	1:500	Immunofluorescence
Goat anti-Mouse IgG (H+L) Secondary Antibody, Alexa Fluor® 488 conjugate	Invitrogen #A-11001	1:500	Immunofluorescence

Table S4. Primers and Oligos

1. Oligos used for in vitro mutagenesis

Gene name	Mutation site	Primers	Sequence (5'-3')
Tet2	S97A	Forward	CCAGAGAGAGATGGTTCAGCGACTGTGCGTTTTATCCC
		Reverse	GGGATAAAACGCACAGTCGCTGAACCATCTCTCTCTGG
Tet2	S97E	Forward	AGCCCAGAGAGAGATGGTTCCTCGACTGTGCGTTTTATCCCTC
		Reverse	GAGGGATAAAACGCACAGTCGAGGAACCATCTCTCTCTGGGCT

2. Oligos used for CRISPR/Cas9 mediated knock-in and knock-out

Gene name		Primers	Sequence (5'-3')
Tet2	sgRNA	Forward	CACCGAAAGTGCCAACAGATATCC
		Reverse	AAACGGATATCTGTTGGCACTTTC
Tet2	Genotyping	Forward	TGAGCCTGTATCCAAACC
		Reverse	GCTAACTCTGGCAAACAC
AMPKα2	sgRNA	Forward	CACCGACAGGCATATGGTTGTCCAT
		Reverse	AAACATGGACAACCATATGCCTGTC
AMPKα2	Genotyping	Forward	GCTGACTCCTCCAAAACATTGTGC
		Reverse	AGGCCAGTGTGAACTGCAATCAACC
Pax7 enhancer	sgRNA-1	Forward	CACCGTTGCGCCGAGACCTTCTGAT
		Reverse	AAACATCAGAAGGTCTCGGCGCAAC
Pax7 enhancer	sgRNA-2	Forward	CACCGAGCTCCAAGCACCTTAGGT
		Reverse	AAACACCTAAGGTGCTTGGAGCTC
Pax7 enhancer	sgRNA-3	Forward	CACCGATGATGTCTGTCAAGTTCGT
		Reverse	AAACACGAACTTGACAGACATCATC
Pax7 enhancer	sgRNA-4	Forward	CACCGAGCAAGTTGTATTTTTCGGG
		Reverse	AAACCCCGAAAAATACAACTTGCTC
Pax7 enhancer	Genotyping1	Forward	GTCCATTCATCCGCTTGTTC
		Reverse	ATGGGCTAGGATGGCAAGTG

Pax7 enhancer	Genotyping2	Forward	ACCAATGTACCTAGCACAGGC
		Reverse	AAGGCTGCAGATGAATGGTGG

3. RT-qPCR Primers

Gene name	Primers	Sequence (5'-3')
Tet1	Forward	TGAAGCTCAAACATCAAGCA
	Reverse	GTACCTCCATCACAGTCAC
Tet2	Forward	AGCGGAGCCCAAGAAAGCCA
	Reverse	CGAAAGCTGCGGTTGTGCTGT
Tet3	Forward	CCTGCGGTGCCTCCTTCTCC
	Reverse	TCCGGAGCACCTCCTCCTCC
Gapdh	Forward	ACCACAGTCCATGCCATCAC
	Reverse	CACCACCCTGTTGCTGTAGCC
Myog	Forward	GAGATCCTGCGCAGCGCCAT
	Reverse	CCCCGCCTCTGTAGCGGAGA
Myf6/MRF4	Forward	GTGGACCCCTACAGCTACAAACC
	Reverse	TGGAAGAAAGGCGCTGAAGAC
Myod1	Forward	TCTGGAGCCCTCCTGGCACC
	Reverse	CGGGAAGGGGGAGAGTGGGG
Myf5	Forward	AAACTCCGGGAGCTCCGCCT
	Reverse	GGCAGCCGTCCGTCATGTCC
18sRNA	Forward	TGCTGTCCCTGTATGCCTCT
	Reverse	TGTAGCCACGCTCGGTCA
Pax7	Forward	GCTACCAGTACAGCCAGTATG
	Reverse	GTCACTAAGCATGGGTAGATG

Full Length Gels and Blots
Zhang T, et al. Phosphorylation of TET2 by AMPK is indispensable in myogenic differentiation

Fig. 1c

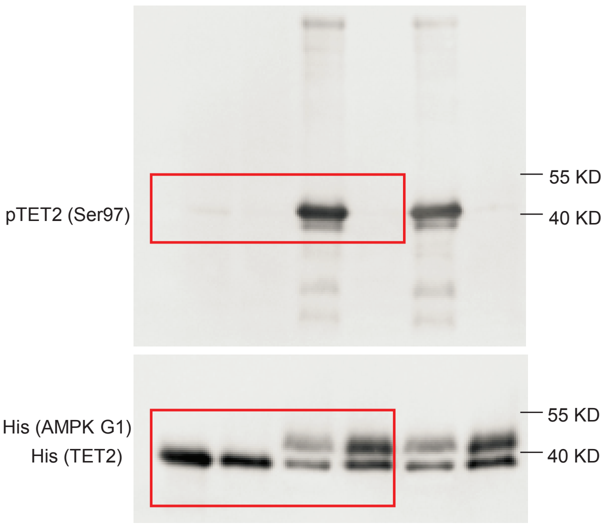

Fig. 1d

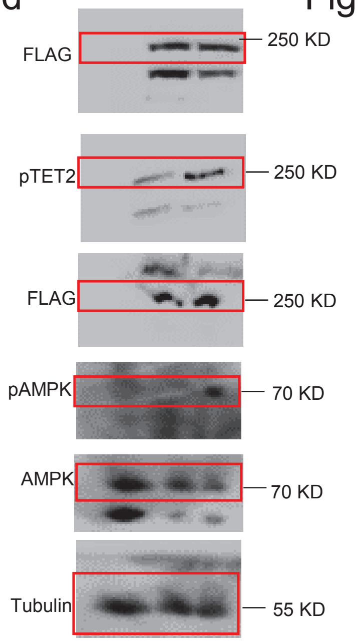

Fig. 1e

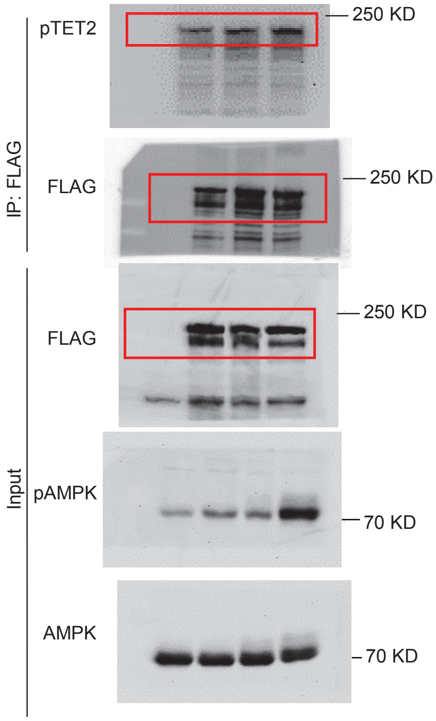

Fig. 1f

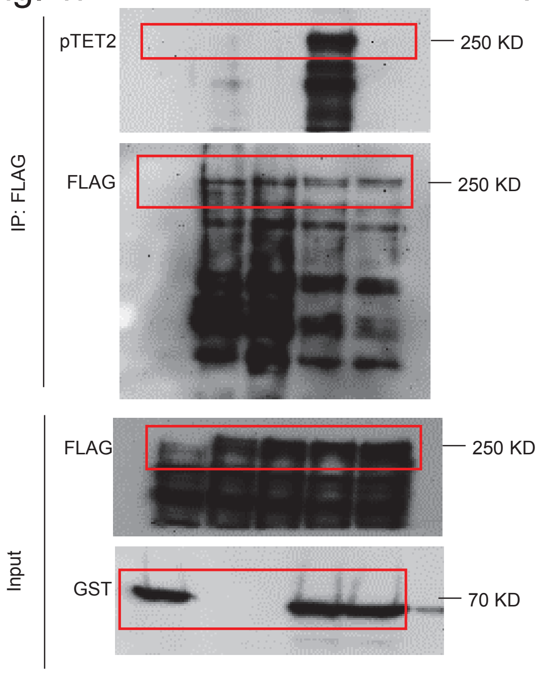

Fig. 1g

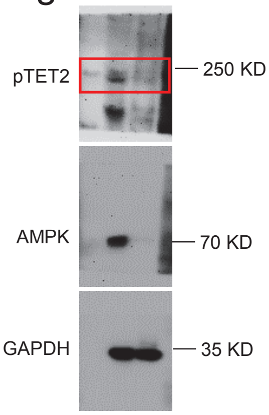

Fig. 1h

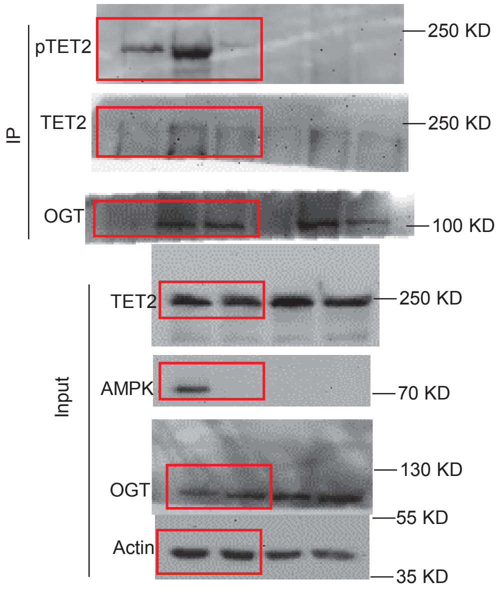

Fig. 2b

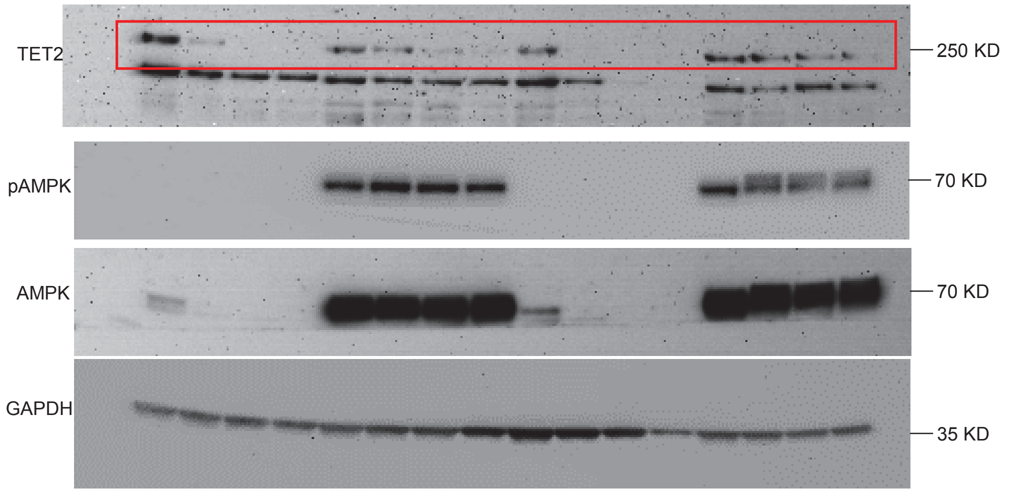

Fig. 2c

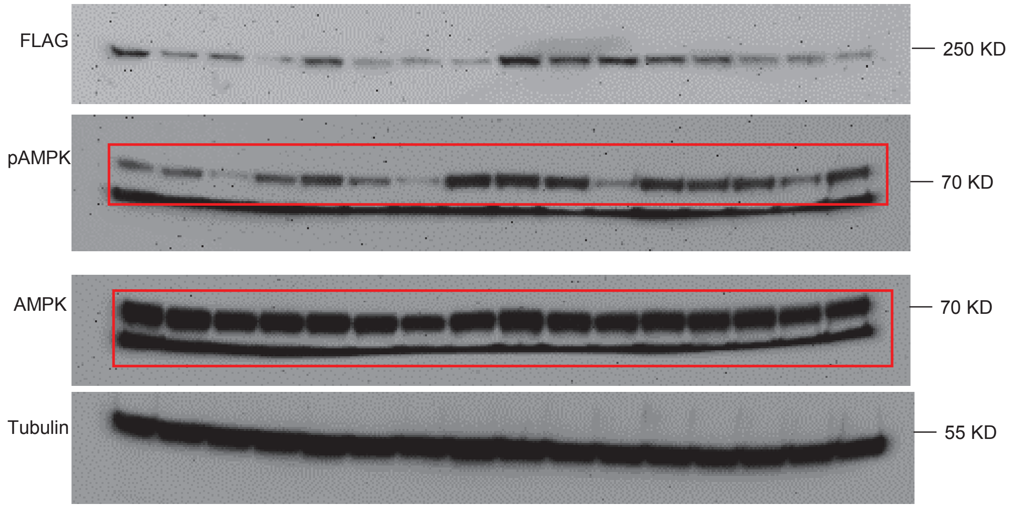

Fig. 2d

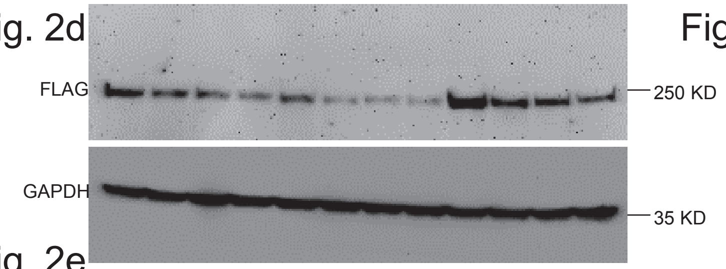

Fig. 2e

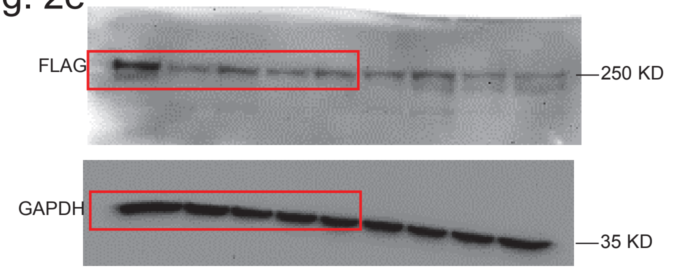

Fig. 2f

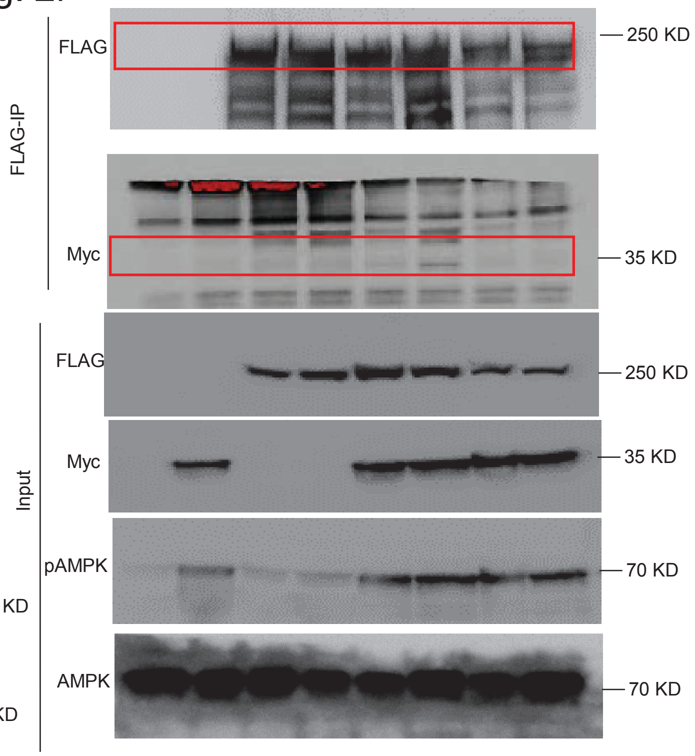

Fig. 2g

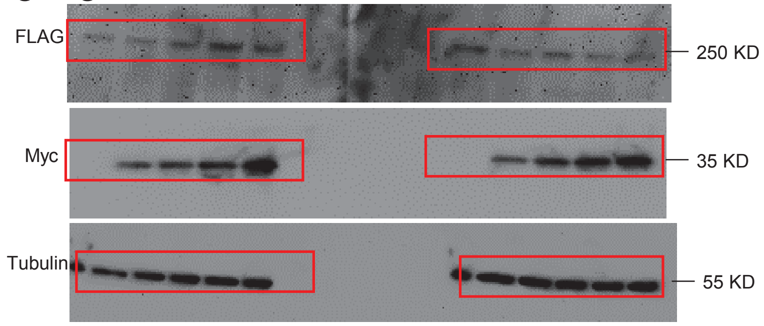

Fig. 3b

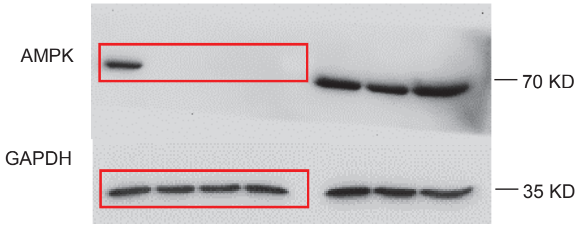

Fig. 3d

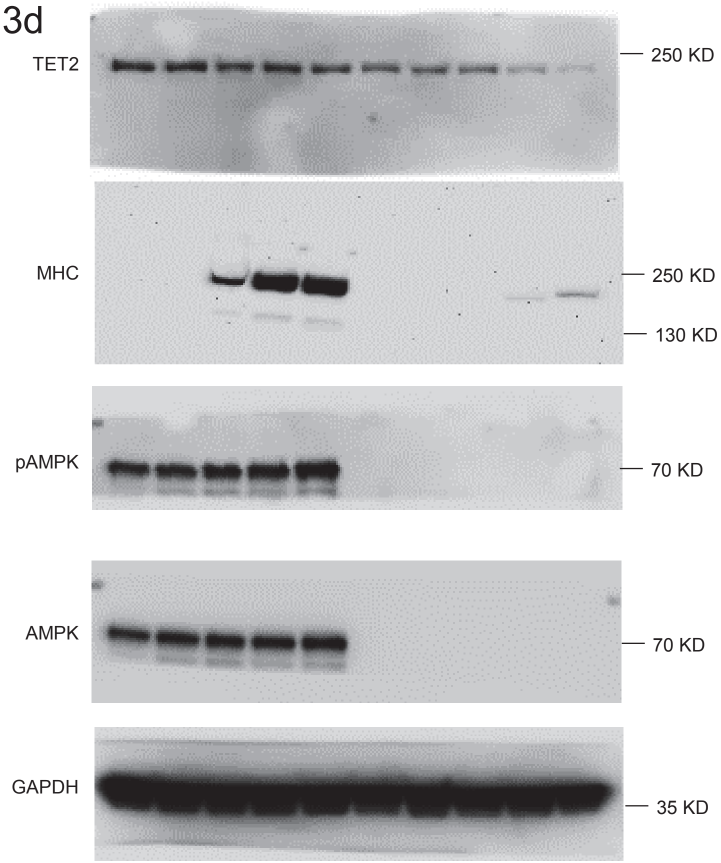

Fig. 6d

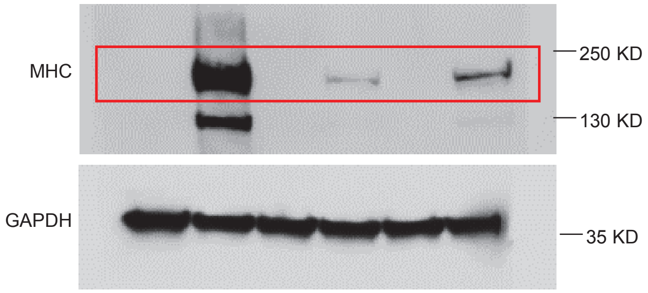

Supplement: Supplementary file 1 — Additional file 1. Fig. S1. SDS-PAGE analysis of the recombinant N-terminus of murine TET2 (aa 1-181) and its mutant. Fig. S2. AMPK knockout impaired the differentiation of C2C12 cells. Fig. S3. Gene ontology analysis of upregulated genes between AMPK-KO and wild-type C2C12 cells at myoblast- (differentiation d0, A) or myotube- stage (differentiation d8, B). Fig. S4. Increased DNA methylation at a potential intragenic enhancer of Pax7. Fig. S5. CRISPR/Cas9-mediated deletion of the Pax7 intragenic enhancer in C2C12 cells. Fig. S6. Knocking in (KI) the pS97E mutation of Tet2 in AMPK-/- C2C12 cells. Fig. S7. S97E mutation of TET2 partly rescues the differentiation defect of the AMPK-/- C2C12 cells. Fig. S8. Increased myosin heavy chain (MHC) expression in AMPK-/- C1C12 cells rescued with TET2 harboring S97E. Table S1. ELISA analysis of pTET2 [Ser99 (h); Ser97(m)] antibodies*. Table S3. Antibodies. Table S4: Primers and Oligos. [file 13072_2019_281_MOESM1_ESM.pdf]
